# Disrupted brain network topology and structure-function coupling in non-specific low back pain

**DOI:** 10.1162/IMAG.a.1412

**Published:** 2026-10-26

**Authors:** Lionel Butry, Frederick Junker, Sam Vickery, Daniel L. Belavy, Rebekka Döding, Katja Ehrenbrusthoff, Maia Angelova, Bjoern Buehring, Bernadette Fitzgibbon, Chandan Karmakar, Clint T. Miller, Patrick J. Owen, Tobias L. Schulte, Scott D. Tagliaferri, Guy Trudel, Jessica Van Oosterwijck, Hans-Joachim Wilke, Elena K. Enax-Krumova, Lara Schlaffke

**Affiliations:** Ruhr University Bochum, BG University Hospital Bergmannsheil, Department of Neurology, Bochum, Germany; Fachbereich Pflege-, Hebammen- und Therapiewissenschaften (PHT), Hochschule Bochum, Bochum, Germany; Institute of Exercise Training and Sport Informatics, German Sport University Cologne, Cologne, Germany; Sir Peter Rigby Digital Futures Institute, Aston University, Birmingham, United Kingdom; Bergisches Rheuma-Zentrum, Krankenhaus St Josef, Wuppertal, Germany; Ruhr University Bochum, Rheumazentrum Ruhrgebiet, Bochum, Germany; School of Medicine and Psychology, College of Health and Medicine, The Australian National University, Canberra, Australia; School of Information Technology, Deakin University, Geelong, Australia; Institute for Physical Activity and Nutrition (IPAN), School of Exercise and Nutrition Sciences, Deakin University, Geelong, Australia; Eastern Health Emergency Medicine Program, Melbourne, Australia; Eastern Health Clinical School, Monash University, Melbourne, Australia; Ruhr University Bochum, St. Josef-Hospital, Katholisches Klinikum Bochum, Department of Orthopaedics and Trauma Surgery, Bochum, Germany; The National Centre of Excellence in Youth Mental Health, Orygen, Parkville, Australia; Centre for Youth Mental Health, University of Melbourne, Melbourne, Australia; Bone and Joint Research Laboratory, Ottawa Hospital Research Institute, Ottawa, Canada; Department of Medicine, The Ottawa Hospital, Ottawa, Canada; Department of Cellular and Molecular Medicine, University of Ottawa, Ottawa, Canada; Spine, Head and Pain Research Unit Ghent, Department of Rehabilitation Sciences, Faculty of Medicine and Health Sciences, Ghent University, Ghent, Belgium; Pain in Motion International Research Consortium, Brussels, Belgium; Institute of Orthopaedic Research and Biomechanics, Ulm University Medical Centre, Ulm, Germany; Department of Information Technology, FH Dortmund, University of Applied Sciences and Arts, Dortmund, Germany; Ruhr University Bochum, St. Josef-Hospital, University Hospital of Pediatrics and Adolescent Medicine, Bochum, Germany

**Keywords:** graph theory, musculoskeletal pain, functional connectivity, structural connectivity, magnetic resonance imaging

## Abstract

Central nervous system mechanisms contribute to non-specific low back pain (LBP), with widespread alterations in brain function and structure reported. It remains unclear how these alterations propagate to brain network topology and structure-function coupling. In this cross-sectional study, 282 participants with non-specific LBP (mean ± standard deviation, pain intensity 4.3 ± 1.7 [0–10 numerical rating scale]; current pain duration 54 ± 105 weeks) and 71 pain-free controls underwent resting-state functional and diffusion-weighted MRI to derive functional and structural connectomes. Subgroups were analyzed by pain status during scanning and pain duration. The main graph-theory analysis revealed that participants with non-specific LBP had lower segregation (*d* = 0.39–0.45, *P* = 0.009–0.019) in the whole-brain functional network and higher integration (*d* = 0.30–0.38, *P* = 0.005–0.027) in the functional ventral attention network. Subgroup analysis showed that participants with chronic non-specific LBP had lower segregation (*d* = 0.48, *P* = 0.002) and higher integration (*d* = 0.45, *P* = 0.004) within the structural default mode network. By modeling functional connectivity using structural communication predictors in a multivariable linear regression, we identified structure-function decoupling in participants with non-specific LBP across the whole-brain (*d* = 0.25–0.28, *P* = 0.026–0.041), ventral attention (*d* = 0.28–0.32, *P* = 0.005–0.048), and default mode network (*d* = 0.22–0.24, *P* = 0.031–0.046), alongside complex regional patterns of both decoupling and hypercoupling. Explorative partial correlation analyses associated disability, rather than pain intensity, with differences in brain network topology and structure-function decoupling. These findings advance the mechanistic understanding of the severity and persistence of non-specific LBP, showing disrupted bottom-up attentional processing and self-referential circuitry, resulting in large-scale structure-function decoupling.

## 1 Introduction

Non-specific low back pain (LBP) accounts for approximately 90% of LBP cases and places a major burden on patients and healthcare systems (Ferreira et al., 2023; Maher et al., 2017). It is characterized by the absence of a clearly identifiable underlying pathology (Maher et al., 2017). In approximately 30% of patients, symptoms persist or recur despite the lack of ongoing peripheral tissue damage (Kongsted et al., 2016), suggesting that mechanisms beyond lumbar nociception contribute to its phenotype. Accordingly, mounting evidence suggests that changes in central pain processing play an important role in non-specific LBP (Tagliaferri et al., 2022; Zhu et al., 2024).

Various pain conditions, including non-specific LBP, show functional and structural neuroplastic changes, particularly when pain persists over prolonged periods (Bautin et al., 2025; Brandl et al., 2022; Fiúza-Fernandes et al., 2025; Kuner & Flor, 2016). These changes affect brain regions such as the thalamus, anterior cingulate cortex, and prefrontal cortex, implicated in sensory-discriminative, affective-motivational, and cognitive-evaluative processes. This distributed involvement highlights that pain is not encoded by isolated regions but emerges from coordinated activity across brain networks (Garcia-Larrea & Bastuji, 2018; Legrain et al., 2011; Wager et al., 2013). In this context, region-specific approaches may provide an incomplete account of pain-related brain alterations, motivating the investigation of pain from a network perspective. Commonly altered large-scale brain networks in chronic musculoskeletal pain include the default mode network (DMN), ventral attention network (VAN), and somatomotor network (SMN) (Bishop et al., 2018; Dhanaraj et al., 2025; Fiúza-Fernandes et al., 2025).

Brain network topology, referring to the organizational architecture of interconnected brain regions, is of particular interest as it naturally shapes the ability of the brain to transfer information (Moon et al., 2015), and can be non-invasively assessed using MRI. It can be examined using both functional connectivity, which captures pairwise statistical relationships between regional activity patterns, and structural connectivity, which represents the underlying white matter architecture (Bullmore & Sporns, 2009). Brain network topology can be quantified with graph theory in terms of integration and segregation, reflecting the efficiency of global information transfer and the degree of specialized processing within submodules (Rubinov & Sporns, 2010). Alterations in brain network topology may, therefore, provide insights into maladaptive or compensational information processing mechanisms that contribute to non-specific LBP.

A recent systematic review of 27 functional and 17 structural topology studies reported, in its meta-analyses, low-certainty evidence for resting-state functional, but not structural, alterations in whole-brain network topology across chronic pain conditions (Butry et al., 2025). This suggests a disruption in the relationship between structural and functional connectivity. As the anatomical scaffold of the brain constrains its functional repertoire, structure-function coupling (SFC) is used to quantify the extent to which neural activity follows the underlying white matter architecture (Fotiadis et al., 2024). Structure-function decoupling has been described in several neurological (Koubiyr et al., 2019; Zarkali et al., 2021; Z. Zhang et al., 2011) and psychiatric (Yao et al., 2024; R. Zhang et al., 2019) disorders and recently also in three chronic pain conditions, including bone metastasis pain, trigeminal neuralgia, and postherpetic neuralgia (Fotiadis et al., 2024; Li et al., 2025; J. Zhang et al., 2026; J. Zheng et al., 2025), highlighting its potential as a comprehensive multimodal indicator of brain network dysfunction. However, whether regional alterations propagate to brain network topology and structure-function decoupling in people with non-specific LBP remains unclear.

To address these gaps, the present study aimed to: (i) characterize differences in structural and functional brain network topology of the whole brain and of large-scale brain networks in patients with non-specific LBP; (ii) investigate SFC; and (iii) explore how these differences relate to clinical self-report measures. At the whole-brain level, we hypothesized lower segregation in functional topology, specifically reflected in local efficiency, no structural topology abnormalities, and lower SFC. No directional hypotheses were formulated on the level of large-scale brain networks.

## 2 Methods

This cross-sectional study was nested within the “Predictive Evidence Driven Intelligent Classification Tool for Low Back Pain (PREDICT-LBP)” project (Belavy et al., 2023). The project was preregistered at the German Clinical Trial Register (DRKS00030518), and ethical approval was obtained according to the Declaration of Helsinki from the local ethics committee of the Medical Faculty of the Ruhr University Bochum (23-7829-BR). The STROBE reporting guideline was followed to ensure transparency (von Elm et al., 2007). A graphical summary of the methods is provided in Figure 1, and results on neural markers of lumbar two-point discrimination and estimation from part of the cohort have been reported previously (Butry, Angelova, et al., 2026).

**Fig. 1.**
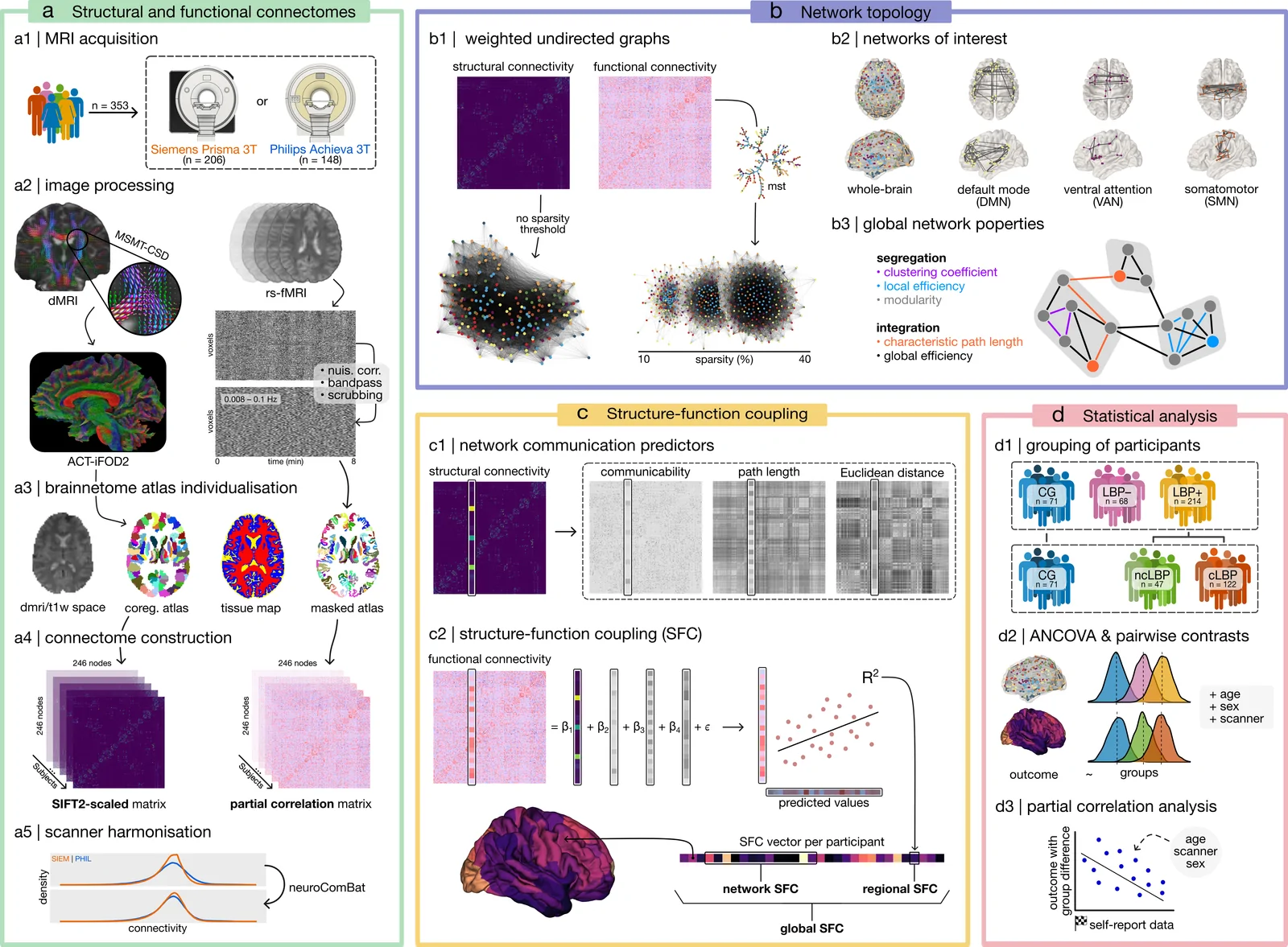
Graphical summary of the methods. (a) Diffusion-weighted MRI (dMRI) and resting-state functional MRI (rs-fMRI) were used to compute structural and functional connectomes for each participant. NeuroComBat was applied to the connectomes to mitigate scanner bias; (b) Global network properties were derived from weighted undirected graphs of functional and structural connectivity for each network of interest; (c) Structure-function coupling per participant was defined as the *R^2^* value of a multivariable linear regression with network communication predictors; (d) Participants were grouped into pain-free control group (CG), LBP without pain during scanning (LBP−), LBP with pain during scanning (LBP+), non-chronic LBP (ncLBP), and chronic LBP (cLBP). Group comparisons were performed using ANCOVA and post-hoc contrasts, controlling for age, sex, and scanner. Explorative partial correlation analyses of the brain metrics and self-report data were conducted, controlling for age, sex, and scanner. ACT-iFOD2, anatomically constrained tractography with second-order integration over fiber orientation distribution algorithm; mst, minimum spanning tree; MSMT-CSD, multi-shell multi-tissue constrained spherical deconvolution; SIFT2, spherical-deconvolution informed filtering of tractograms; t1w, T1-weighted.

### 2.1 Participants

Participants with non-specific LBP and pain-free controls, recruited for the PREDICT-LBP project between August 2023 and December 2025, were included (Belavy et al., 2023). Recruitment was conducted via flyers in medical and public locations across the Ruhr Area, Germany, and through social media and online advertisements. Adults aged 18–55 years were eligible, reflecting the typical onset and peak burden of LBP within this age range (Hartvigsen et al., 2018).

LBP was defined as pain between the costal arch and gluteal fold, with or without leg pain, regardless of pain duration (Belavy et al., 2023).

General exclusion criteria were (Belavy et al., 2023): (i) diagnosed non-degenerative specific spinal conditions (e.g., vertebral fracture), spinal nervous system damage (e.g., radiculopathy), or severe degenerative spinal conditions (e.g., spondylolisthesis); (ii) previous spine or brain surgery; (iii) prior major head trauma; (iv) diagnosed neurological or major psychiatric disorder; (v) pregnancy or ≤6 months postpartum; and (vi) absolute MRI contraindications such as metal implants or severe claustrophobia.

Additional exclusions for pain-free controls were (Belavy et al., 2023): (i) current spinal pain affecting function; (ii) LBP lasting >24 hours within the past year; (iii) any LBP-related work absence; and (iv) any prior healthcare consultation for LBP. Controls were aimed to match to LBP participants by age and sex.

Eligibility was determined by an online questionnaire followed by a structured telephone interview. No further neurological assessments for neuropathic pain were conducted. All eligible participants were included in the study. Participants provided written informed consent, received 100€ compensation, and were instructed not to disclose their pain status to the study staff during data acquisition.

Functional resting-state activity is potentially influenced by the presence of pain during scanning. Therefore, participants with LBP were subdivided into two groups according to self-reported presence of pain during scanning, obtained immediately after MRI acquisition: Participants experiencing pain during scanning (LBP+) and participants without pain during scanning (LBP−). For subgroup analysis, the LBP+ group was stratified by current pain duration at assessment into chronic (cLBP; >3 months) and non-chronic (ncLBP; ≤3 months). Only the LBP+ group was included in the subgroup analysis to ensure that pain duration corresponded to the pain state during scanning.

### 2.2 Demographic and clinical self-report measures

During online screening, average LBP intensity during the past 7 days and LBP duration (weeks) were assessed. Participants had two on-site visits: one for clinical assessments and another for MRI scanning.

During the on-site assessment, age, sex at birth (female/male), average LBP intensity during the past 7 days, current LBP intensity, current LBP duration (weeks), and medication use were self-reported and body mass index (BMI) was assessed. The following validated questionnaires were administered: Sensitization Inventory (CSI) (Neblett et al., 2013), Oswestry Disability Index (ODI) (Fairbank & Pynsent, 2000), Tampa Scale of Kinesiophobia (TSK) (Rusu et al., 2014), PROMIS-Anxiety, and PROMIS-Depression (Cella et al., 2007). Average (past 7 days) and current LBP intensity at assessment was queried by an 11-point numeric rating scale (0 = “no pain”; 10 = “highest imaginable pain”). Medication use was assessed by free-text and later categorized by type (non-steroidal anti-inflammatory drugs [NSAIDs], metamizole, paracetamol, weak opioids, strong opioids, cannabinoids, muscle relaxants, others). Current pain duration and current LBP intensity at assessment were collected only when LBP was present at the time of questionnaire administration.

At the MRI visit, presence of pain during scanning was assessed. Additionally, at the Prisma MRI site, current LBP intensity during scanning was assessed after MRI using a 100 mm visual analogue scale (0 = “no pain”; 100 = “highest imaginable pain”) and later reported in cm.

### 2.3 MRI acquisition

Participants were scanned using either a 3.0T Achieva MRI system (Philips Healthcare, Best, The Netherlands) or a 3T Magnetom Prisma MRI system (Siemens, Healthineers, Munich, Germany) with a 32- or 64-channel head coil, respectively. A T1-weighted (T1w) scan was acquired as an anatomical reference, followed by resting-state fMRI and multi-shell dMRI. Sequence details are provided in Supplementary Table S2. During resting-state fMRI, participants were instructed to close their eyes, stay awake, and let their minds wander without focusing on anything specific. Owing to identified data quality issues with the Siemens dMRI sequence (Supplementary Fig. S1), acceleration factors were reduced, resulting in two variants: *SIEM-1* and *SIEM-2* (Supplementary Table S1).

### 2.4 Image processing

Details of the MRI preprocessing pipelines were described in previous work by our group (Butry, Angelova, et al., 2026; Butry, Thomä, et al., 2026). Briefly, using fMRIPrep (v23.2.0), T1w images were non-uniformity corrected, skull-stripped, and segmented into cerebrospinal fluid (CSF), white matter, and grey matter (Esteban et al., 2019). Resting-state fMRI data were slice-time corrected and coregistered to each participant’s native T1w space. Confounding time series were derived from preprocessed data, except head-motion parameters, which were estimated before any spatiotemporal filtering. Each time series was band-pass filtered (0.008–0.1 Hz) and denoised based on a benchmarking study using the ‘scrubbing.5’ strategy, including nuisance regression of 24 motion and eight white matter and CSF parameters, as well as scrubbing of volumes with excessive motion (FD >0.5 mm) (Wang et al., 2024). Lastly, signals were z-score standardized, and datasets with >30% of volumes scrubbed were excluded.

The dMRI pipeline followed the structural connectivity framework implemented in MRtrix3 (v3.0.3) (Tournier et al., 2019). Initial preprocessing included denoising and correction for Gibbs ringing, motion artefacts, eddy currents, inhomogeneities, and bias field. As data from the *SIEM-1* dMRI sequence were prone to slice artefacts, additional preprocessing with *dStripe*, an established constrained neural network, was performed to mitigate these artefacts (Supplementary Fig. S1) (Pietsch et al., 2021). Fiber orientation distributions (FODs) were estimated using a multi-shell multi-tissue constrained spherical deconvolution (MSMT-CSD) diffusion model (Jeurissen et al., 2014). Probabilistic tractography (*iFOD2*) with 5,000,000 streamlines, 1 mm step size, 45° angle, 4 mm minimum track length, 250 mm maximum track length, 0.05 FOD amplitude cutoff, and backtracking enabled, was used within the anatomically-constrained tractography (ACT) framework, with the grey-to-white-matter interface as both the seed and terminal region (Smith et al., 2012). Accordingly, preprocessed T1w images were coregistered to each participant’s native dMRI space.

Visual quality assessment was performed on raw and preprocessed MRI data. Images were inspected for artefacts, misalignments, and other pipeline errors. Additionally, MRIQC (v25.1.0) was applied to raw fMRI and dMRI data (Esteban et al., 2017). A summary of MRIQC’s image quality metrics is provided in Supplementary Figures S2 and S3.

### 2.5 Connectome construction

The Brainnetome atlas defined regions of interest (ROIs), comprising 246 regions (36 subcortical, 210 cortical; Supplementary Table S2) (Fan et al., 2016). The atlas was coregistered to each participant’s native T1w and dMRI space using b-spline nonlinear transformation with nearest-neighbour interpolation (*antsRegistrationSyNQuick.sh*, ANTs) for constructing functional and structural connectomes, respectively. For the nodes of the functional connectomes, the coregistered atlas was further masked using the participant’s grey matter mask, derived from fMRIPrep’s discrete tissue segmentation output.

To obtain biologically meaningful estimates of structural connectivity, probabilistic tractograms were filtered with *SIFT2* (*tcksift2*, MRtrix3), assigning a weight to each streamline to match its underlying FOD amplitudes (Smith et al., 2015). Structural connectivity was defined as the sum of weights of all streamlines connecting a pair of atlas regions (*tck2connectome*, MRtrix3) (Smith et al., 2015). The resulting structural connectivity values were proportional to the effective cross-sectional area of white matter axons linking the corresponding ROIs (Smith et al., 2015).

For functional connectivity, Nilearn’s *ConnectivityMeasure*, a Python-based tool that computes pairwise statistics between regional mean blood-oxygen-level-dependent (BOLD) signal time series, was applied to obtain a partial correlation matrix for each participant. Partial correlations were chosen because they show higher correspondence to structural connectivity and better predictive performance than the commonly used Pearson correlation (Liu et al., 2025). Thus, functional connectivity represents the direct pairwise correlation between the brain activity of two ROIs during rest.

### 2.6 Harmonization

Structural and functional connectivity can vary systematically across scanners, potentially compromising comparability but these scanner-dependent effects can be mitigated by feature-based harmonization techniques (Butry, Thomä, et al., 2026). Accordingly, prior to subsequent analysis structural and functional connectivity were harmonized separately using the established *ComBat* algorithm as implemented in the R package *neuroCombat* (v1.0.14) (Fortin et al., 2018). Scanner was modeled as the batch variable, with group, age, and sex as covariates using default parameters. For fMRI, scanner was a binary variable (PHIL; SIEM) and for dMRI it explicitly included the protocol change (PHIL; SIEM-1; SIEM-2).

### 2.7 Graph theory analysis

#### 2.7.1 Graph construction

Individual weighted graphs were constructed from harmonized structural and functional connectomes, represented as undirected adjacency matrices where each value represents the strength of the pairwise connection (edge weight) between ROIs (nodes) (Bullmore & Sporns, 2009). The full graph with 246 nodes contains 245 × 246 / 2 = 30,135 unique edges. Subgraphs of large-scale brain network, including DMN (36 × 36), SMN (33 × 33) and VAN (22 × 22), were induced from the full graph of each participant. Connection length matrices were obtained via reciprocal weight-to-length transformation ($Length=\frac{1}{Weight}$ ), representing the cost of traversing an edge and were used in the calculation of distance-based global network properties.

No sparsity threshold was applied to the dense structural graphs, as removing weak connections provides no practical utility (Civier et al., 2019). Functional graphs, which contain many spurious connections, were thresholded using a combined minimum spanning tree (MST) and density approach (Fornito et al., 2016). An MST was first constructed from the connection length matrix to preserve the strongest connections without fragmentation, followed by adding edges in descending weight order until reaching a predefined sparsity level. Given the impact of graph density on network topology and the lack of a gold standard (Fornito et al., 2013; Wijk et al., 2010), a sparsity range of 10–40% in 5% increments was chosen, consistent with prior pain research (Butry et al., 2025).

#### 2.7.2 Global network properties

Weighted global network properties were computed for functional and structural graphs across the whole-brain network and within DMN, SMN and VAN subgraphs using the R package *igraph* (v2.2.2) (Csárdi et al., 2026). For functional graphs, metrics were calculated across the density range and summarized as the area under the curve (AUC) for group comparisons (Bassett et al., 2012).

Global network properties reflected measures of both integration and segregation (Rubinov & Sporns, 2010). Clustering coefficient (CC), modularity (Q), and local efficiency (E_loc_) measure network segregation, reflecting local connectivity, modular organization, and the efficiency of information transfer within local neighbourhoods. Characteristic path length (L) and global efficiency (E_glob_) quantify network integration, capturing average shortest paths and the efficiency of information transfer across the entire network.

### 2.8 Structure-function coupling

Building on prior approaches to structure-function coupling (SFC) (Fotiadis et al., 2024; Liu et al., 2022, 2025), a multivariable linear regression model with network communication and geometric predictors was employed to evaluate the relationship between structural and functional connectivity. Here, structural connectivity itself was additionally included as a predictor alongside shortest path length, communicability, and Euclidean distance, enabling the model to capture direct anatomical connectivity alongside indirect communication:

$$FC_{i}\;=\;\beta_{0}\;+\;\beta_{1}SC_{i}\;+\beta_{2}PL_{i}\;+\beta_{3}COM_{i}\;+\beta_{4}ED_{i}\;+\varepsilon_{i}$$ (1)

where $FC_{i}$ was the vector of functional connectivity from node *i* to all others and $SC_{i}$, $PL_{i}$, $COM_{i}$, and $ED_{i}$ were the corresponding vectors of structural connectivity, shortest path length, communicability, and Euclidean distance, respectively. The four-predictor model was validated against the conventional three-predictor model (excluding $SC_{i}$), yielding increased explained variance while the resulting SFC estimates remained highly correlated between the two models (Supplementary Fig. S4). The variance inflation factor was < 5 for all predictors, suggesting non-collinearity (Supplementary Table S3). SFC of each region was quantified using adjusted R-squared ($R_{adj}^{2}$), a measure of goodness of fit. Higher $R_{adj}^{2}$ values indicate stronger structural constraints on functional connectivity. Global SFC was the mean across all regions and network SFC the mean within each large-scale network (Fotiadis et al., 2024).

Network communication metrics were derived from each participant’s structural connection length matrix. Shortest path length refers to the minimum distance needed to travel between two nodes (Rubinov & Sporns, 2010). Communicability measures the number of possible walks linking two nodes (Seguin et al., 2023). The geometric predictor, Euclidean distance, was calculated as the distance in MNI space between region centroids. The metrics were computed using the R packages *igraph* (v2.2.2) and *brainGraph* (v3.1.1).

### 2.9 Statistical analysis

Statistical analyses were performed separately for the main groups (CG, LBP+, LBP−) and subgroups (CG, ncLBP, cLBP) using R (v4.5.2, www.r-project.org). The significance level was set at α = 0.05. Participants with missing values were excluded from the respective analysis. Data distribution and variance homogeneity of sample characteristics were assessed using QQ-plots and Levene’s test, respectively. Group differences in sample characteristics were tested using appropriate omnibus tests (one-way ANOVA, Kruskal–Wallis-test, or χ^2^-test). Depending on the omnibus test, either pairwise Tukey’s HSD, Dunn test, or Wilcoxon test was performed with false discovery rate (FDR) correction (R package: *rstatix* v0.7.2).

As the population distribution of many global network properties is often unknown a priori, nonparametric tests are recommended (Fornito et al., 2016). Group differences were assessed using permutation-based ANCOVAs with 50,000 permutations (R package: *permuco* v1.1.3) (Frossard & Renaud, 2021), with FDR correction. Permutation-based linear models with pairwise contrasts were utilized as post-hoc tests. Group differences in structure-function coupling were analyzed by linear models and pairwise contrasts. Age, sex, and scanner were set as covariates of no interest in all models. Scanner had two levels (*PHIL*; *SIEM*) in fMRI and three levels (*PHIL*; *SIEM-1*; *SIEM-2*) in dMRI, considering the dMRI protocol change. Effect sizes for ANCOVA were reported as partial eta-squared ($\eta^{2}$) and group comparisons used Cohen’s *d* with 95% confidence intervals (*d* [95% CI]), calculated with the R package *effectsize* v1.0.1 (Ben-Shachar et al., 2020). Effect size magnitudes were not interpreted against conventional thresholds, as these are arbitrary and may not be appropriate for brain-derived metrics (Tagliaferri et al., 2024).

Two explorative analyses were conducted: (i) group differences in region-wise SFC using the same procedures as above; and (ii) explorative correlation analysis between significant variables of the group difference analyses and clinical self-report measures. Partial Spearman correlations were used to adjust for age, sex, and scanner. No corrections for multiple testing were applied in the explorative analyses, warranting replication of results in independent samples.

## 3 Results

### 3.1 Participants

The final study sample consisted of 353 participants, distributed across three groups: LBP+ (*n* = 214), LBP− (*n* = 68), and CG (*n* = 71). The participant flowchart is presented in Figure 2. In median, 29 (*Q1*: 18, *Q3*: 48) days past between screening and on-site visit and 9 (*Q1*: 5, *Q3*: 16) days between on-site clinical and MRI visit. The distribution of the study groups across the two scanners and the dMRI protocols is reported in Supplementary Table S4.

**Fig. 2.**
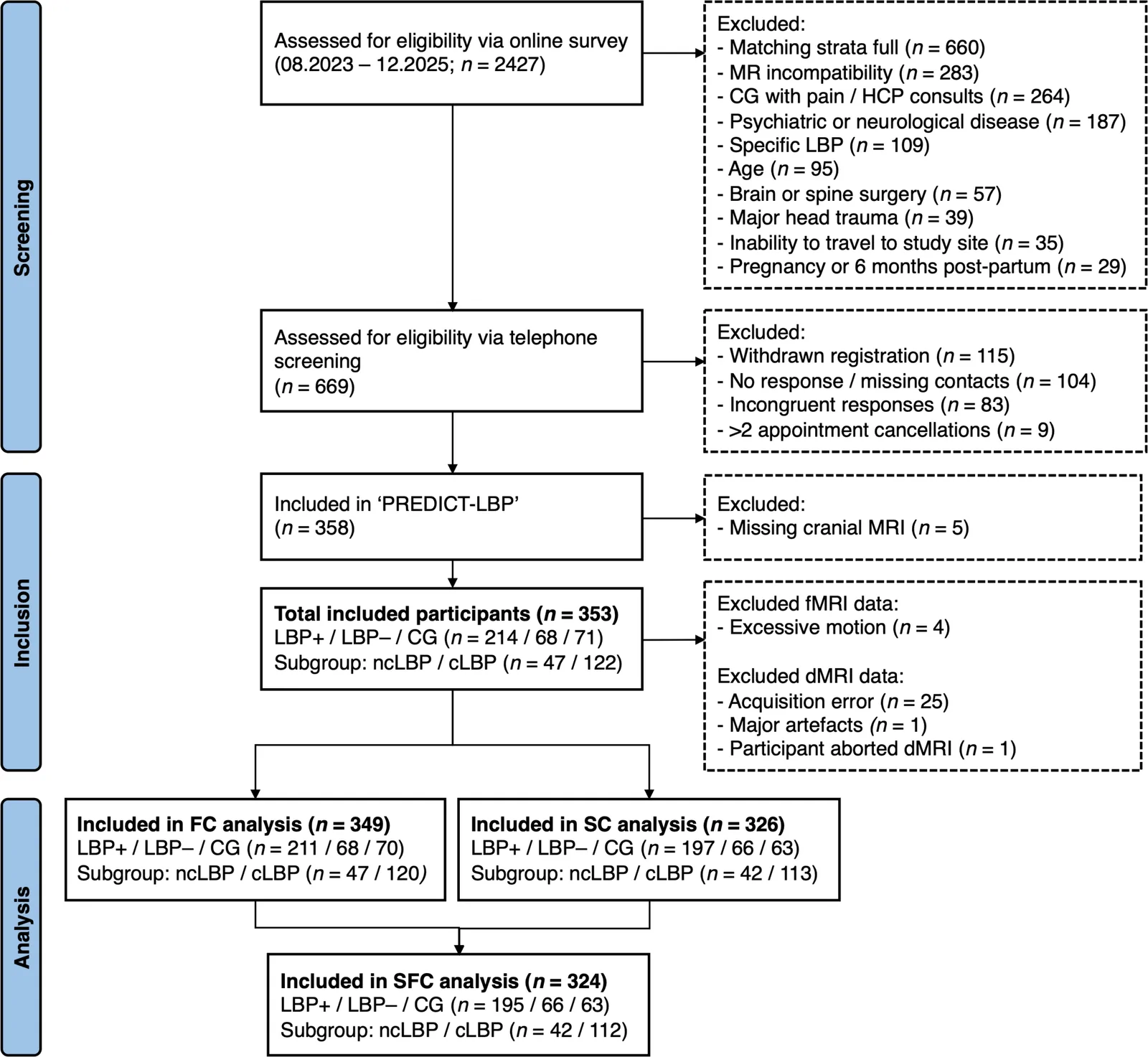
Flowchart of participant enrolment. cLBP, chronic low back pain; CG, pain-free control group; dMRI, diffusion-weighted MRI; FC, functional connectome; fMRI, functional MRI; HCP, health care practitioner; LBP+, low back pain with pain during scanning; LBP−, low back pain without pain during scanning; ncLBP, non-chronic low back pain; SC, structural connectome; SFC, structure-function coupling.

A detailed description of the sample is provided in Table 1. Age and sex were distributed equally in the three groups. In the LBP+ group, the pain intensity was low to moderate, and 57% had current pain lasting longer than 3 months and was therefore categorized as chronic pain. Pain medication usage was low in both LBP groups. Both LBP+ and LBP− had higher anxiety (PROMIS), symptoms of central sensitization (CSI), disability (ODI), and kinesiophobia (TSK) scores compared to the control group. Comparing LBP+ and LBP−, the latter showed lower disability (ODI) and less symptoms of central sensitization (CSI). LBP+ had higher depression scores (PROMIS) than controls. Head motion (FD), controlled for scanner, did not differ significantly between LBP+, LBP−, and CG for fMRI (ANCOVA: *P* = 0.899, post-hoc: *P* = 0.795–0.824) or dMRI (ANCOVA: *P* = 0.056, post-hoc: *P* = 0.111–0.848).

**Table 1.** Characteristics of the study sample stratified by reported pain during scanning.

	Main LBP groups			Statistics	
Characteristics	LBP+ (n = 214)	LBP− (n = 68)	CG (n = 71)	Global^a^	Post hoc
Age (years)	36 (25, 44)	35 (28, 43)	31 (23, 41)	0.059	–
BMI (kg/m^2^)	24.8 (22.5, 29.3)	23.7 (22.2, 27.1)	23.3 (21.1, 25.7)	**0.005**	CG vs LBP+ (**)
Sex (female)	128 (60%)	38 (56%)	40 (56%)	0.788	–
Cur. LBP intensity during scanning (VAS in cm)^b^	1.9 (0.9, 3.1)	0.0 (0.0, 0.0)	–	–	**<0.001**
Cur. LBP intensity at assessment (NRS)^c^	3.0 (2.0, 5.0)	3.0 (2.0, 4.0)	–	–	**0.031**
Avg. LBP intensity at assessment (NRS)^c^	4.0 (3.0, 5.0)	4.0 (2.8, 5.0)	–	–	0.214
Avg. LBP intensity at screening (NRS)^d^	4.0 (3.0, 5.0)	3.0 (2.0, 4.0)	–	–	**0.001**
Pain duration reported at screening (weeks)^d,e^	30 (10, 150) [3, 1502]	20 (13, 41) [4, 667]	–	–	**0.001**
Cur. LBP duration at assessment (weeks)^c^	52 (12, 250) [1, 3650]	35 (6, 88) [1, 572]	–	–	–
Cur. LBP duration at assessment (categorical)^c ^					
*- acute (<6 weeks)*	18 (8%)	6 (9%)	–	–	–
*- subacute (6–12 weeks)*	29 (14%)	17 (25%)	–	–	–
*- chronic (>12 weeks)*	122 (57%)	5 (7%)	–	–	–
Medication: *- NSAIDs*	15 (7%)	0 (0%)	2 (3%)	–	–
*- Metamizole*	4 (2%)	0 (0%)	0 (0%)	–	–
*- Paracetamol*	1 (0.5%)	0 (0%)	0 (0%)	–	–
*- Weak opioids*	4 (2%)	1 (1%)	0 (0%)	–	–
*- Strong opioids*	1 (0.5%)	0 (0%)	0 (0%)	–	–
*- Cannabinoids*	2 (1%)	0 (0%)	0 (0%)	–	–
*- Muscle relaxants*	2 (1%)	0 (0%)	0 (0%)	–	–
*- Other*	36 (17%)	16 (24%)	8 (11%)	–	–
ODI	14 (10, 22)	8 (4, 14)	0 (0, 2)	**<0.001**	CG vs LBP− (***) CG vs LBP+ (***) LBP− vs LBP+ (***)
TSK	21 (18, 25)	20 (17, 24)	16 (14, 20)	**<0.001**	CG vs LBP− (***) CG vs LBP+ (***)
CSI	33 (25, 43)	29.5 (20, 35)	16 (11, 23)	**<0.001**	CG vs LBP− (***) CG vs LBP+ (***) LBP− vs LBP+ (**)
PROMIS Anxiety	7.0 (5.0, 9.0)	6.5 (5.0, 9.0)	5.0 (4.0, 7.0)	**0.005**	CG vs LBP− (**) CG vs LBP+ (**)
PROMIS Depression	6.0 (4.0, 9.0)	5.5 (4.0, 8.0)	5.0 (4.0, 7.0)	**0.012**	CG vs LBP+ (*)

Values in median (Q1, Q3) [Min, Max] or n (%). Bold text indicates significant results.

a Kruskal–Wallis-Test: age, BMI, ODI, CSI, PROMIS Anxiety, PROMIS Depression; χ^2^-Test: sex; one-way ANOVA: TSK.

b Missing data (LBP+: n = 78, LBP−: n = 32).

c Missing data (LBP+: n = 45, LBP−: n = 40).

d Missing data (LBP−: n = 6).

e Adjusted for time between screening and assessment.

*, *P* < 0.05; **, *P* < 0.01; ***, *P* < 0.001.

avg, average; BMI, body mass index; cur, current; CSI, Central Sensitization Inventory; CG, control group; cLBP, chronic low back pain; ncLBP, non-chronic low back pain; NRS, numeric rating scale; NSAIDs, non-steroidal anti-inflammatory drugs; ODI, Oswestry Disability Index; TSK, Tampa Scale of Kinesiophobia.

Characteristics of the subsamples of LBP+, stratified by current pain duration, are summarised in Table 2. A total of 45 participants in the LBP+ group were excluded due to missing data on current LBP duration at assessment, leaving 169 participants for the chronicity subgroup analyses. Participants with chronic LBP (cLBP) showed no difference in current and average pain intensity, disability (ODI), kinesiophobia (TSK), anxiety (PROMIS), and depression (PROMIS), but higher symptoms of central sensitization (CSI) compared to non-chronic LBP (ncLBP).

**Table 2.** Characteristics of the subsamples of LBP+ stratified by current pain duration (>3 months; ≤3 months).

	Subgroups of LBP+			Statistics	
Characteristics	ncLBP (n = 47)	cLBP (n = 122)	CG (n = 71)	Global^a^	Post hoc
Age (years)	32 (26, 41)	37 (27, 45)	31 (23, 41)	**0.017**	CG vs cLBP (*)
BMI	25.9 (23.8, 29.9)	24.2 (22.2, 29.1)	23.3 (21.1, 25.7)	**0.005**	CG vs ncLBP (**)
Sex (female)	28 (60%)	74 (61%)	40 (56%)	0.829	–
Cur. LBP intensity during scanning (VAS in cm)^b^	1.4 (0.9, 2.5)	2.5 (1.1, 3.9)	–	–	0.100
Cur. LBP intensity at assessment (NRS)	4.0 (3.0, 4.5)	3.0 (2.0, 5.0)	–	–	0.925
Avg. LBP intensity at assessment (NRS)	4.0 (3.0, 5.0)	4.0 (3.0, 6.0)	–	–	**0.027**
Avg. LBP intensity at screening (NRS)	4.0 (3.0, 5.0)	4.0 (3.0, 6.0)	–	–	0.323
Pain duration reported at screening (weeks)^c^	14 (8, 22) [3, 1004]	70 (34, 217) [5, 1502]	–	–	**<0.001**
Cur. LBP duration at assessment (weeks)	6 (4, 8) [1, 12]	107 (46, 359) [13, 3650]	–	–	**<0.001**
Cur. LBP duration at assessment (categorical):					
*- acute (<6 weeks)*	18 (38%)	0 (0%)	–	–	–
*- subacute (6–12 weeks)*	29 (62%)	0 (0%)	–	–	–
*- chronic (>12 weeks)*	0 (0%)	122 (100%)	–	–	–
Medication:				–	–
*- NSAIDs*	2 (4%)	11 (9%)	2 (3%)	–	–
*- Metamizole*	1 (2%)	3 (2%)	0 (0%)	–	–
*- Paracetamol*	0 (%)	1 (1%)	0 (0%)	–	–
*- Weak opioids*	1 (2%)	3 (2%)	0 (0%)	–	–
*- Strong opioids*	0 (%)	1 (1%)	0 (0%)	–	–
*- Cannabinoids*	0 (%)	1 (1%)	0 (0%)	–	–
*- Muscle relaxants*	0 (%)	1 (1%)	0 (0%)	–	–
*- Other*	3 (4%)	27 (22%)	8 (11%)	–	*–*
ODI	14 (8, 18)	16 (12, 26)	0 (0, 2)	**<0.001**	CG vs ncLBP (***) CG vs cLBP (***)
TSK	20 (18, 25)	22 (18, 25)	16 (14, 20)	**<0.001**	CG vs ncLBP (***) CG vs cLBP (***)
CSI	30 (23, 38)	37 (27, 46)	16 (11, 23)	**<0.001**	CG vs ncLBP (***) CG vs cLBP (***) ncLBP vs cLBP (*)
PROMIS Anxiety	6.0 (4.5, 9.0)	7.0 (5.0, 9.0)	5.0 (4.0, 7.0)	**0.002**	CG vs cLBP (**)
PROMIS Depression	5.0 (4.0, 8.5)	7.0 (5.0, 9.0)	5.0 (4.0, 7.0)	**0.002**	CG vs cLBP (**)

Values in median (Q1, Q3) or n (%). Bold text indicates significant results.

a Kruskal–Wallis-Test: age, BMI, ODI, CSI, PROMIS Anxiety, PROMIS Depression; χ^2^-Test: sex; one-way ANOVA: TSK.

b Missing data (ncLBP: n = 16, cLBP: n = 44).

c Adjusted for time between screening and assessment.

*, *P* < 0.05; **, *P* < 0.01; ***, *P* < 0.001.

avg, average; BMI, body mass index; cur, current; CSI, Central Sensitization Inventory; CG, control group; cLBP, chronic low back pain; ncLBP, non-chronic low back pain; NRS, numeric rating scale; NSAIDs, non-steroidal anti-inflammatory drugs; ODI, Oswestry Disability Index; TSK, Tampa Scale of Kinesiophobia.

The excluded participants from functional topology analysis (*n* = 4), structural topology analysis (*n* = 27) and structure-function coupling analysis (*n* = 29) did not differ statistically from the included participants regarding group, age, and sex. Thus, the missing data were unlikely to introduce bias into the results or meaningfully reduce statistical power.

### 3.2 Structural and functional network topology

No statistically significant group differences were observed in global network properties of the whole-brain structural connectome, or with the structural DMN, SMN and VAN among CG, LBP−, and LBP+ (Supplementary Table S5).

In the whole-brain functional connectome or within the functional DMN and SMN, no significant group differences were observed in global network properties (Supplementary Table S6). In contrast, within the functional topology of the VAN, participants in the LBP+ group had higher global efficiency (*d* [95% CI] = 0.30 [0.07, 0.54], *P* = 0.005) and lower characteristic path length (−0.34 [−0.10, −0.56], *P* = 0.013) compared to controls (Fig. 3a). Global efficiency and characteristic path length did not differ between LBP− and LBP+, nor between LBP− and controls.

**Fig. 3.**
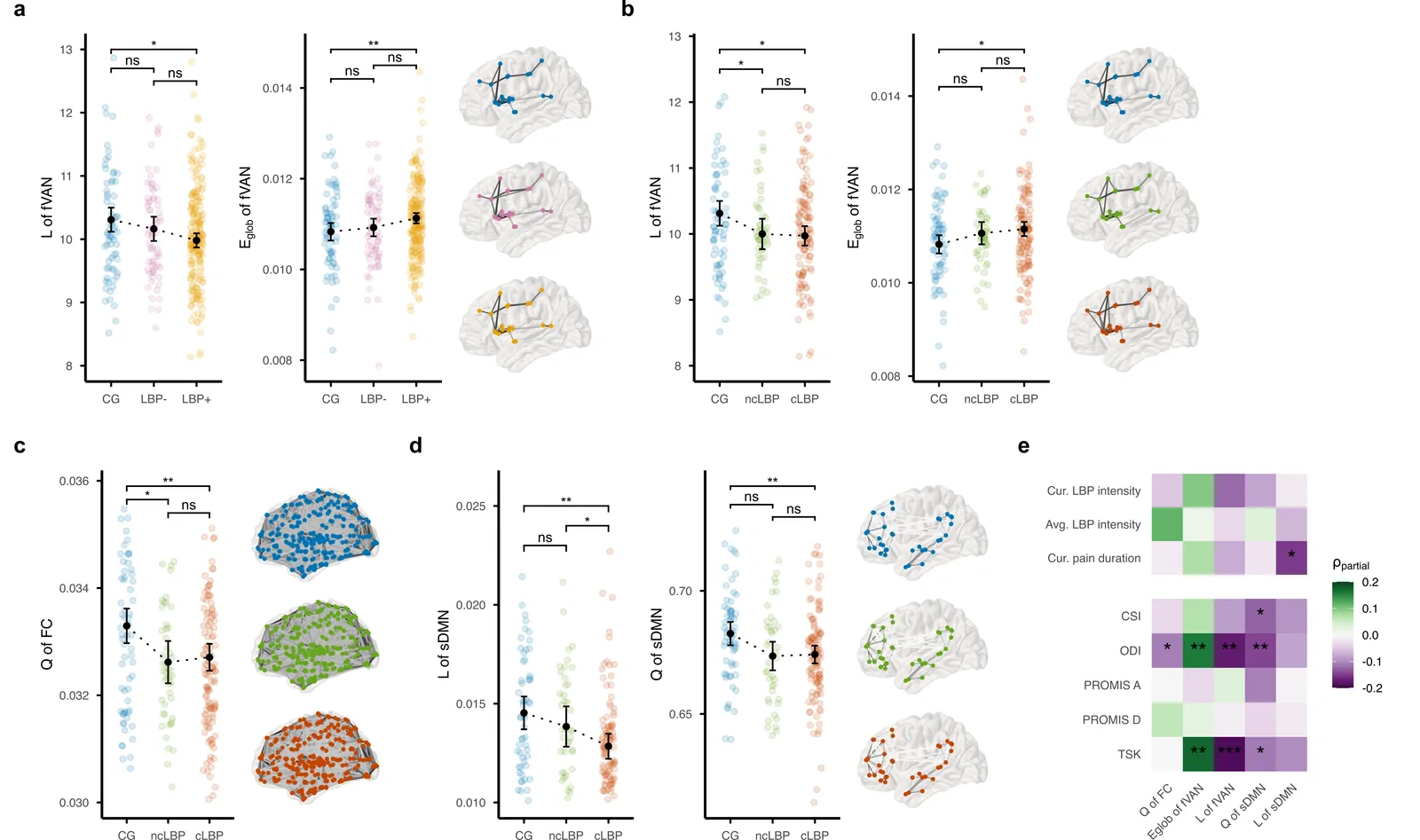
Differences in structural and function brain network topology in LBP. (a) Significant differences in global network properties between CG, LBP−, and LBP+. Colored points reflect unadjusted values, and black points represent marginal means with 95% confidence intervals. (b–d) Significant differences in global network properties between CG, ncLBP, and cLBP; ordered by large-scale brain network. (e) Partial Spearman correlations between clinical self-report measures and altered global network properties. Lower half includes all participants. Upper half only includes LBP groups. (Info) Brain graph plots shown at 15% sparsity of group average connectivity matrix. Darker edges represent higher connectivity. cLBP, chronic low back pain; CG, control group; CSI, central sensitization inventory; Eglob, global efficiency; FC, whole-brain functional connectivity; fVAN, functional ventral attention network; L, characteristic path length; LBP−, low back pain group without pain during scanning; LBP+, low back pain group with pain during scanning; ncLBP, non-chronic low back pain; ns, *P* > 0.05; ODI, Oswestry Disability Index; PROMIS A, PROMIS anxiety; PROMIS D, PROMIS depression; Q, modularity; sDMN, structural default mode network; TSK, Tampa Scale of Kinesiophobia; *, *P* < 0.05; **, *P* < 0.01; ***, *P* < 0.001.

In subgroup analyses stratified by current pain duration, the CG demonstrated a lower characteristic path length of the functional VAN relative to cLBP (*d* [95% CI] = 0.37 [0.08, 0.66], *P* = 0.013) and ncLBP (0.38 [0.04, 0.79], *P* = 0.028) but cLBP and ncLBP showed no difference (Fig. 3b). Additionally, global efficiency of the functional VAN was higher in cLBP compared to controls (0.35 [0.06, 0.64], *P* = 0.013) but did not differ between ncLBP and CG nor cLBP (Fig. 3b). The whole-brain functional connectome showed lower modularity in the ncLBP (−0.45 [−0.08, −0.83], *P* = 0.019) and cLBP (−0.39 [−0.10, −0.68], *P* = 0.009) group compared to the CG (Fig. 3c). In the structural DMN, the characteristic path length was lower in cLBP compared to CG (−0.45 [−0.15, −0.76], *P* = 0.004) and ncLBP (−0.37 [−0.04, −0.70], *P* = 0.027) but showed no difference between CG and ncLBP (Fig. 3d). Additionally, lower modularity of the structural DMN was observed in cLBP compared to CG (−0.48 [−0.17, −0.78], *P* = 0.002) but no differences between ncLBP and CG nor ncLBP (Fig. 3d). Details of the subgroup analyses are provided in Supplementary Tables S7 and S8.

An exploratory partial correlation analysis controlling for age, sex, and scanner showed several associations between global network properties and clinical self-report measures (Fig. 3e). Higher modularity of the whole-brain functional connectome was associated with lower disability (ODI, ρ = −0.109, *P* = 0.044). Higher global efficiency of the functional VAN was associated with higher kinesiophobia (TSK, 0.173, *P* = 0.001) and disability (ODI, 0.158, *P* = 0.003). Higher characteristic path length of the functional VAN was associated with lower kinesiophobia (TSK, −0.188, *P* < 0.001) and disability (ODI, −0.174, *P* = 0.001). Higher modularity of the structural DMN was associated with lower kinesiophobia (TSK, −0.112, *P* = 0.045), disability (ODI, −0.146, *P* = 0.009), and symptoms of central sensitization (CSI, −0.127, *P* = 0.023). Lower characteristic path length of the structural DMN was associated with longer current pain duration (−0.152, *P* = 0.042).

### 3.3 Structure-function coupling

Across all groups, global SFC was on average 0.035 ± 0.009, with visual inspection of regional SFC indicating higher SFC in unimodal cortical regions and lower SFC in transmodal association regions (Supplementary Fig. S5).

When testing for group differences between CG, LBP−, and LBP+, weaker global SFC was demonstrated for LBP− compared to CG (*d* [95% CI] = −0.25 [−0.03, −0.47], *P* = 0.026) but no differences between LBP+ and CG, nor LBP− (Fig. 4a). In the DMN, weaker SFC was shown in LBP− (−0.24 [−0.02, −0.46], *P* = 0.031) and LBP+ (−0.22 [0.00, −0.45], *P* = 0.046) relative to controls, while LBP− and LBP+ showed no difference (Fig. 4a). A similar pattern was observed in the VAN, with weaker SFC in LBP− (−0.32 [−0.09, −0.54], *P* = 0.005) and LBP+ (−0.28 [−0.06, −0.50], *P* = 0.014) compared to CG but not between LBP− and LBP+ (Fig. 4a). While a significant main group effect was indicated in SMN-SFC ($\eta^{2}$ = 0.023, *P*_FDR_ = 0.027), the post-hoc tests showed no significant differences (Fig. 4a).

**Fig. 4.**
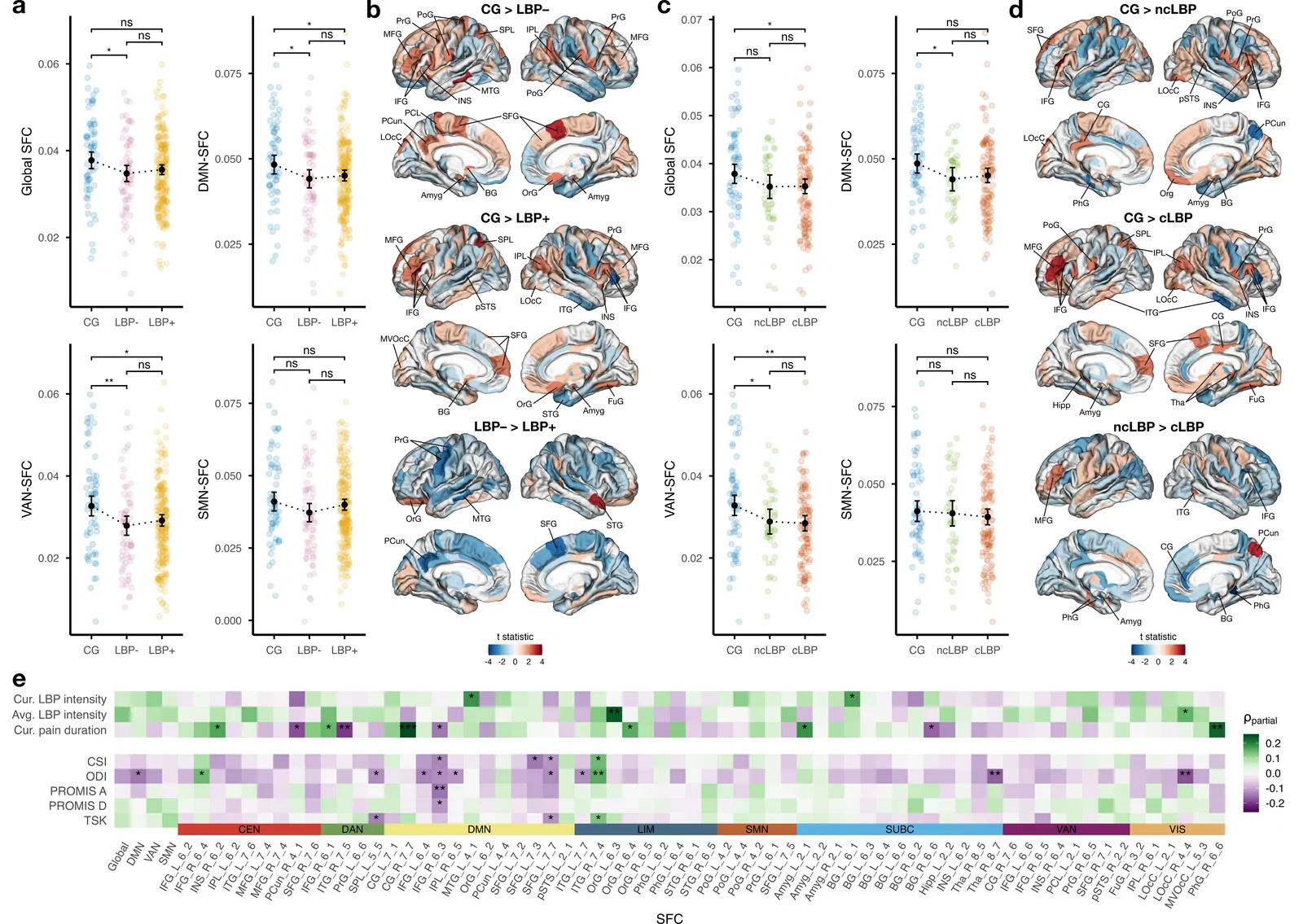
Differences in structure-function coupling (SFC) in LBP. (a) Group differences in global and network-wise SFC between CG, LBP−, and LBP+. Colored points reflect unadjusted values, and black points and error bars represent marginal means with 95% confidence intervals. (b) Group differences in region-wise SFC between CG, LBP−, and LBP+. Brainnetome regions were projected to surface space and coloured by t statistic per contrast. Annotated regions have *P* < 0.05. Subcortical regions may be anatomically distorted. (c, d) Equivalent to panel a and b but for CG, ncLBP, and cLBP. (e) Partial spearman correlations controlling for age, sex, and scanner between clinical self-report measures and altered SFC variables. Lower half includes all participants. Upper half includes only LBP groups. cLBP, chronic low back pain; CEN, central executive network; CG, control group; CSI, central sensitization inventory; DAN, dorsal attention network; DMN, default mode network; LBP−, low back pain without pain during scanning; LBP+, low back pain with pain during scanning; LIM, limbic network; ncLBP, non-chronic low back pain; ns, *P* > 0.05; ODI, Oswestry Disability Index; PROMIS A, PROMIS anxiety; PROMIS D, PROMIS depression; SMN, somatomotor network; SUBC, subcortical network; TSK, Tampa Scale of Kinesiophobia; VAN, ventral attention network; VIS, visual network; *, *P* < 0.05; **, *P* < 0.01; ***, *P* < 0.001.

The explorative analysis of regional SFC differences between CG, LBP−, and LBP+ revealed widespread changes, with affected regions distributed across all large-scale brain networks (Fig. 4b; Supplementary Fig. S6a). Compared to the control group, LBP− exhibited weaker SFC across all regions reaching significance (*n* = 24; 5 in SUBC; 4 in VAN, SMN, CEN; 3 in DMN; 2 in DAN; 1 in VIS, LIM). Comparing LBP+ to the control group, 20 regions had weaker SFC (5 in DMN; 4 in VIS; 3 in VAN, SUBC, CEN; 1 in LIM, DAN) and four regions had stronger SFC (2 in LIM; 1 in DMN, CEN). Between the two patient groups, LBP+ and LBP− differed in eight regions, with LBP− showing lower SFC than LBP+ in five regions (2 in DMN; 1 in VAN, SMN, DAN) and higher SFC in the remaining three (2 in LIM; 1 in DMN). It should be noted that none of the regional SFC differences survived FDR correction.

In subgroup analyses stratified by current pain duration, cLBP showed weaker global SFC compared to CG (*d* [95% CI] = −0.28 [−0.01, −0.55], *P* = 0.044), but no differences were observed between ncLBP and CG, nor cLBP (Fig. 4c). In the DMN, weaker SFC was demonstrated in ncLBP (−0.28 [−0.01, −0.55], *P* = 0.041), but no differences between cLBP and CG, nor ncLBP (Fig. 4c). In the VAN, weaker SFC was demonstrated in ncLBP (−0.28 [−0.003, −0.55], *P* = 0.047) and cLBP (−0.38 [−0.11, −0.65], *P* = 0.006) compared to controls, but no differences between ncLBP and cLBP (Fig. 4c).

When exploring regional SFC differences in the subgroups stratified by current pain duration, changes remained widespread across all large-scale brain networks (Fig. 4d; Supplementary Fig. S6b). Compared to controls, ncLBP showed lower SFC in 14 regions (4 in DMN; 2 in VAN, CEN, VIS; 1 in SMN, SUBC, DAN, LIM) and higher SFC in three regions (1 in SUBC, LIM, CEN). Compared to CG, cLBP exhibited lower SFC in 23 regions (6 in VAN; 4 in DMN, CEN; 3 in VIS, SUBC; 1 in SMN, LIM, DAN) and higher SFC in three regions (1 in CEN, LIM, SUBC). Between ncLBP and cLBP, the later showed higher SFC in six regions (2 in SUBC; 1 in DMN, DAN, LIM, VIS) and lower SFC in five (2 in CEN; 1 in SUBC, LIM, DAN). Note, that none of the regional SFC differences survived FDR correction.

The exploratory partial correlation analysis controlling for age, sex, and scanner identified several associations between SFC and clinical self-report measures (Fig. 4e). Among the self-report measures examined, disability (ODI) showed the greatest number of associations with SFC (*n* = 11), followed by current pain duration (*n* = 10). Higher disability (ODI) was associated with weaker DMN-SFC (ρ = −0.141, *P* = 0.011) and weaker SFC across eight brain regions (ρ = −0.113 to −0.174, P = 0.002 to 0.042), including the right lateral occipital cortex, right caudal temporal thalamus, bilateral inferior frontal gyrus, left superior frontal gyrus, left superior parietal lobule, left inferior temporal gyrus, and right inferior parietal lobule. Conversely, higher disability (ODI) was also associated with stronger SFC in the right inferior temporal gyrus (ρ = 0.154, P = 0.006) and right inferior frontal gyrus (ρ = 0.137, P = 0.014). Longer current pain duration was associated with stronger SFC in six regions (ρ = 0.147 to 0.252, P ≤ 0.001 to 0.049), including the right cingulate gyrus, right parahippocampal gyrus, left medial amygdala, right insular cortex, right orbital gyrus, and right inferior frontal gyrus. Additionally, longer current pain duration was associated with weaker SFC in four regions (ρ = −0.147 to −0199, P = 0.007 to 0.049), including the right inferior temporal gyrus, right precuneus, right dorsolateral putamen, and right inferior frontal gyrus. Interestingly, higher pain intensity was associated with stronger SFC across four regions (ρ = 0.151 to 0.239, P = 0.001 to 0.044), including the left orbital gyrus, left middle temporal gyrus, left ventral caudate, and right lateral occipital cortex. Spatially, associations were more frequent within the DMN, which exhibited mostly negative associations, whereas no associations were observed in regions of the SMN and VAN.

Detailed SFC values per network and region are additionally provided in Supplementary Tables S9 and S10.

## 4 Discussion

This study investigated structural and functional brain network topology and structure-function coupling (SFC) in 282 participants with non-specific LBP and 71 pain-free controls.

Contrary to our initial hypothesis, non-specific LBP was accompanied by differences in both structural and functional topology. Functional topology differences, particularly in bottom-up attentional processing, may reflect a compensatory response to current pain, while structural disruptions within the default mode network (DMN) may represent a predisposing factor or early consequence. At large scales, structure-function decoupling was observed, though regional patterns revealed both de- and hypercoupling. Disability showed the most consistent associations with brain metrics across all clinical self-report measures.

### 4.1 Abnormal white matter architecture of the DMN: Predisposing factor or early consequence of LBP?

Participants with non-specific LBP had no differences in structural whole-brain topology compared to pain-free controls, suggesting preserved white matter architecture. This aligns with our hypothesis, based on a prior meta-analysis of several chronic pain conditions (Butry et al., 2025). However, within the structural DMN, lower characteristic path length and modularity suggest greater integration and lower segregation in patients with chronic LBP, reflecting less separation between DMN submodules and more efficient configurations of axonal pathways for within-DMN communication. As the DMN has been posited to sustain self-referential and internally directed thought (Menon, 2023), these structural differences may relate to internally focused cognitive processes, including kinesiophobia and pain catastrophizing. This is supported by associations between modularity of the structural DMN and kinesiophobia and disability in our study. Kinesiophobia and pain catastrophizing are common in participants with non-specific LBP and have been repeatedly shown to predict the development of chronic LBP and influence treatment efficacy (Picavet et al., 2002; Wertli et al., 2014; Wood et al., 2023). They have also been linked to reduced grey matter and white matter integrity in the medial prefrontal cortex, a key hub of the DMN (Hubbard et al., 2014; Im et al., 2021; Y. Zhang et al., 2025). Targeting kinesiophobia and pain catastrophizing may, therefore, not only improve treatment efficacy but also potentially influence white matter architecture associated with these maladaptive cognitive processes in chronic non-specific LBP.

Our results suggest a dissociation between the two structural DMN metrics, each showing a distinct pattern of group differences and self-report correlates. For characteristic path length, the cLBP group differed from both ncLBP and CG. Together with the partial correlation between lower characteristic path length and longer current pain duration (ρ = −0.152), this suggests that it may reflect pain persistence rather than a pre-existing vulnerability or rapid structural change. For modularity, ncLBP and cLBP did not differ, while CG showed a notable visual deviation from both groups; however, a statistically significant difference was only found between CG and cLBP. It remains unclear whether low modularity of the structural DMN constitutes a predisposing neural marker or an early onset consequence of LBP. The latter is supported by evidence that microstructural white matter changes, measured by diffusion tensor imaging, can occur within hours to weeks due to learning tasks (Sagi et al., 2012; Scholz et al., 2009). Evidence for low structural DMN modularity as a potential risk factor for LBP is partly provided by a longitudinal diffusion tensor imaging study in patients with back pain (Mansour et al., 2013). It concluded that altered structural connectivity, including between DMN regions, may predispose individuals to chronic pain. However, these interpretations require caution due to the cross-sectional design of our study. Nonetheless, our findings advance the understanding of LBP chronification by linking maladaptive cognitive processes, such as kinesiophobia, to measurable differences in structural DMN topology. Future longitudinal studies tracking structural connectivity from acute to chronic LBP, ideally with interventions targeting kinesiophobia or pain catastrophizing, are needed to clarify the directional relationship between structural DMN topology and clinical self-report measures.

### 4.2 Abnormal functional topology in the full connectome and the VAN

Consistent with our hypothesis, based on prior meta-analytic observations in several chronic pain conditions (Butry et al., 2025), our results showed lower segregation of the whole-brain functional connectome in ncLBP and cLBP. Unlike the meta-analysis reporting lower local efficiency (Hedge’s *g* = −0.50) (Butry et al., 2025), present findings showed lower modularity, suggesting that differences primarily affect large-scale functional community structure rather than local network neighbourhoods. Additionally, our findings suggest that low whole-brain functional modularity is characteristic of the pain experience irrespective of its duration, as both ncLBP and cLBP showed the difference, and modularity was associated with disability but not current pain duration.

Focusing on large-scale brain networks, the LBP+ group, particularly cLBP, exhibited lower characteristic path length and higher global efficiency within the functional ventral attention network (VAN), indicating greater network integration. As the VAN mediates bottom-up reorientation toward salient stimuli (Corbetta & Shulman, 2002), greater integration may reflect heightened attentional sensitivity to pain-related or behaviorally relevant signals at rest. Supporting this, greater VAN integration was associated with more pronounced kinesiophobia and disability. As a core region of the VAN, the insula may contribute to the greater VAN integration observed here. Prior chronic pain studies have shown that the insula acquires greater hub status (Kaplan et al., 2019) and show hyperconnectivity with the cingulate cortex (Ichesco et al., 2014) and the DMN (Napadow et al., 2012), all of which were associated with clinical pain scores.

Notably, differences in VAN topology occurred in LBP+ but not LBP−, suggesting that they may relate to the experience of current pain during scanning. Prior experimental studies showed that acutely induced pain enhanced functional connectivity within the VAN, suggesting a compensatory response to pain (Fairclough et al., 2023; Seminowicz & Davis, 2007; W. Zheng et al., 2020). Higher integration of the functional VAN may reflect this compensational reorganization on a topology level. However, pain-during-scanning effects should be interpreted cautiously as the LBP− group differed clinically from the LBP+ group with lower disability (ODI) and symptoms of central sensitization (CSI). Longitudinal studies are needed to establish greater functional VAN integration as a compensatory response to pain, as it cannot be conclusively determined from the current data.

### 4.3 Complex structure-function remodelling linked to LBP

SFC magnitude and spatial patterns were comparable to studies using similar multivariable linear regression models with network communication predictors (Liu et al., 2022; Vázquez-Rodríguez et al., 2019). At the whole-brain level, both LBP− and cLBP groups exhibited weaker global SFC than controls, with a trend toward weaker SFC in LBP+ and ncLBP. However, effects were small and spatially widely distributed as reflected in exploratory regional SFC analysis. Previous studies reported mixed findings: lower global SFC in bone metastasis pain and trigeminal neuralgia (J. Zhang et al., 2026; J. Zheng et al., 2025), whereas no alterations in postherpetic neuralgia (Li et al., 2025). Together, this suggests weak global structure-function decoupling in LBP, potentially dependent on the underlying pain condition.

At the network level, structure-function decoupling of the VAN was observed across all LBP groups compared to controls, with no difference between the LBP groups. The DMN exhibited weaker SFC in LBP+, LBP−, and ncLBP and a trend toward weaker SFC in cLBP. Similar DMN and structure-function decoupling of the salience network was reported in patients with postherpetic neuralgia (Li et al., 2025). Considering altered functional VAN topology and structural DMN topology, weaker VAN-SFC may attribute to altered functional connectivity, whereas weaker DMN-SFC may attribute to altered structural connectivity. VAN-SFC was not associated with any of the assessed clinical self-report measures, suggesting limited clinical relevance. In contrast, weaker DMN-SFC was associated with higher disability, and associations with clinical self-report measures were most frequent in DMN regions. This adds another layer to the ongoing discourse around prioritizing disability over pain intensity in pain science and clinical practice (Ballantyne & Sullivan, 2015; Taylor et al., 2024), motivating further investigations into DMN-SFC in LBP severity.

Interestingly, higher current and average pain intensity were not associated with weaker SFC and were instead associated with stronger SFC in four regions. Further, longer current pain duration was associated with stronger SFC in multimodal regions linked to emotional-evaluative processing, alongside weaker SFC in regions spanning cognitive, motor, and salience-related processes. Together, these findings suggest that associations between pain characteristics and SFC are region-specific and heterogenous, indicating that uniform structure-function decoupling models may not fully capture brain-pain relationships.

### 4.4 Specificity of large-scale brain disruptions to LBP

Pain-related disability rather than pain intensity showed the most consistent associations with network topology and SFC. This raises the possibility that these effects are not specific to LBP but reflect a more general, transdiagnostic correlate of functional impairment shared across brain disorders. For example, common grey matter abnormalities in the DMN have been reported across chronic pain, anxiety, and major depressive disorder (Brandl et al., 2022). While the current findings cannot establish that the observed brain differences are specific to LBP, this does not render them irrelevant. Indeed, Zebhauser et al. (2026) argue that transdiagnostic brain-based biomarkers can still be clinically and mechanistically informative, even in the absence of disorder-specific effects.

### 4.5 Methodological considerations

The current study must be interpreted in the light of several limitations. The targeted age range (18–55 years) may limit generalizability, as the sample skews toward younger individuals with lower pain intensity and disability compared to the national BURDEN 2020 study, which reports stable back pain prevalence across the lifespan and severe or very severe pain in 35% of cases (von der Lippe et al., 2021). Group sizes were uneven, though age and sex were equally distributed in the main groups. Current LBP duration refers to the present pain episode and may therefore not accurately reflect the first onset of LBP in their lifetime, particularly in individuals with intervening pain-free periods. The robustness of the pain duration measures was limited given the discrepancies between pain duration reported at screening and at assessment. Non-pharmacological treatment utilization was not assessed despite its potential influence on brain structure and function. Despite comparable MRI protocols, post-hoc harmonization, and accounting for scanner differences in all models, remaining variability may have reduced statistical power. The cross-sectional design precludes causal interpretation. The Brainnetome atlas provides a validated and unified parcellation scheme for functional and structural connectivity, however it does not include the brainstem and cerebellum, which likely play an important role in LBP (Gilliam et al., 2026; Mao et al., 2025). Static resting-state functional connectivity may have reduced the complexity of dynamic brain activity at rest, potentially obscuring time-varying network interactions. Restricting spontaneous slow oscillations to 0.008–0.1 Hz limits findings to this frequency band. Future studies could explore frequency-dependent mechanisms in pain-related alterations (Gong & Zuo, 2025). No multiple comparison correction was applied to the explorative partial correlation and regional SFC analyses, necessitating replication in independent samples.

### 4.6 Conclusion

The findings advance the mechanistic understanding of how functional and structural brain network topology contribute to the severity and persistence of non-specific LBP, by disrupting bottom-up attentional processing and self-referential circuitry, resulting in large-scale structure-function decoupling. The study provides a neuroimaging framework that longitudinal studies can build upon to clarify causal pathways and subsequently inform more targeted, brain-informed treatment strategies.

## Supplementary Material

Supplementary Material

## Data Availability

The PREDICT-LBP project is ongoing and expected to conclude in April 2027; therefore, the data used in this manuscript cannot yet be shared. Upon completion, the data will be made publicly available per funding agency’s agreement, with a link provided at (https://github.com/lbutry/paper_stfu-topo), where the analysis code is already accessible.
