# Supplementary material for: Disrupted brain network topology and structure-function coupling in non-specific low back pain
Source: Imaging Neurosci (Camb). 2026 Oct 26;4:IMAG.a.1412. doi: 10.1162/IMAG.a.1412 (PMC13710934; doi:10.1162/IMAG.a.1412)
Supplement: Supplementary file 1 [file IMAG.a.1412_supp.pdf]

Supplementary information – Disrupted brain network topology and structure-function coupling in non-specific low back pain

Table of Content

1 Methods – MRI acquisition & Image processing 2
2 Methods – Connectome construction 6
3 Methods – Structure-function coupling 11
4 Results – Participants 12
5 Results – Structural and functional topology 12
6 Results – Structure-function coupling (SFC) 16

1 Methods – MRI acquisition & Image processing

Table S1: *Overview of MRI protocol parameters.*

	Philips Achieva TX 3.0T	Siemens Prisma 3T	
**T1w**			
Pulse sequence	MP-3D GRE	MP-3D GRE	
TR	7.70 ms	1650 ms	
TE	3.60 ms	2.98 ms	
Flip angle	8°	8°	
Matrix size	240×240	256×256	
No. of slices	220	176	
Slice gap	0 mm	0 mm	
Resolution	1×1×1 mm^3	1×1×1 mm^3	
Duration	4 min 1 s	3 min 57 s	
**rs-fMRI**			
Pulse sequence	GE-EPI	GE-EPI	
TR	2500 ms	2500 ms	
TE	30 ms	30 ms	
Flip angle	90°	90°	
Matrix size	80x80	80x80	
No. of slices	47	48	
Slice gap	0 mm	0 mm	
Resolution	3×3×3 mm^3	3×3×3 mm^3	
No. of volumes	190	190	
Acceleration	SENSE: 3	MB: 2, GRAPPA: 4	
Duration	~7 min 55s	~7 min 55 s	
**dMRI**		SIEM-1	SIEM-2
Pulse sequence	SE-EPI	SE-EPI (cmrr_mbep2d_diff)	SE-EPI (cmrr_mbep2d_diff)
TR	10000 ms	3800 ms	8210 ms
TE	91 ms	90 ms	90 ms
Flip angle	90°	90°	90°
Matrix size	112×112	110×110	110×110
No. of slices	61	68	68
Slice gap	0 mm	0 mm	0 mm
Resolution	2×2×2 mm^3	2×2×2 mm^3	2×2×2 mm^3
No. of volumes	117	117	117
Acceleration	SENSE: 2.5	MB: 2, GRAPPA: 3	GRAPPA: 3
B values	0; 1000; 1800; 2500	0; 1000; 1800; 2500	0; 1000; 1800; 2500
Gradient directions	12; 30; 30; 45	12; 30; 30; 45	12; 30; 30; 45

Note. dMRI, diffusion-weighted magnetic resonance imaging; T1w, T1-weighted; rs-fMRI, resting-state functional magnetic resonance imaging; TE, echo time; TR, repetition time.

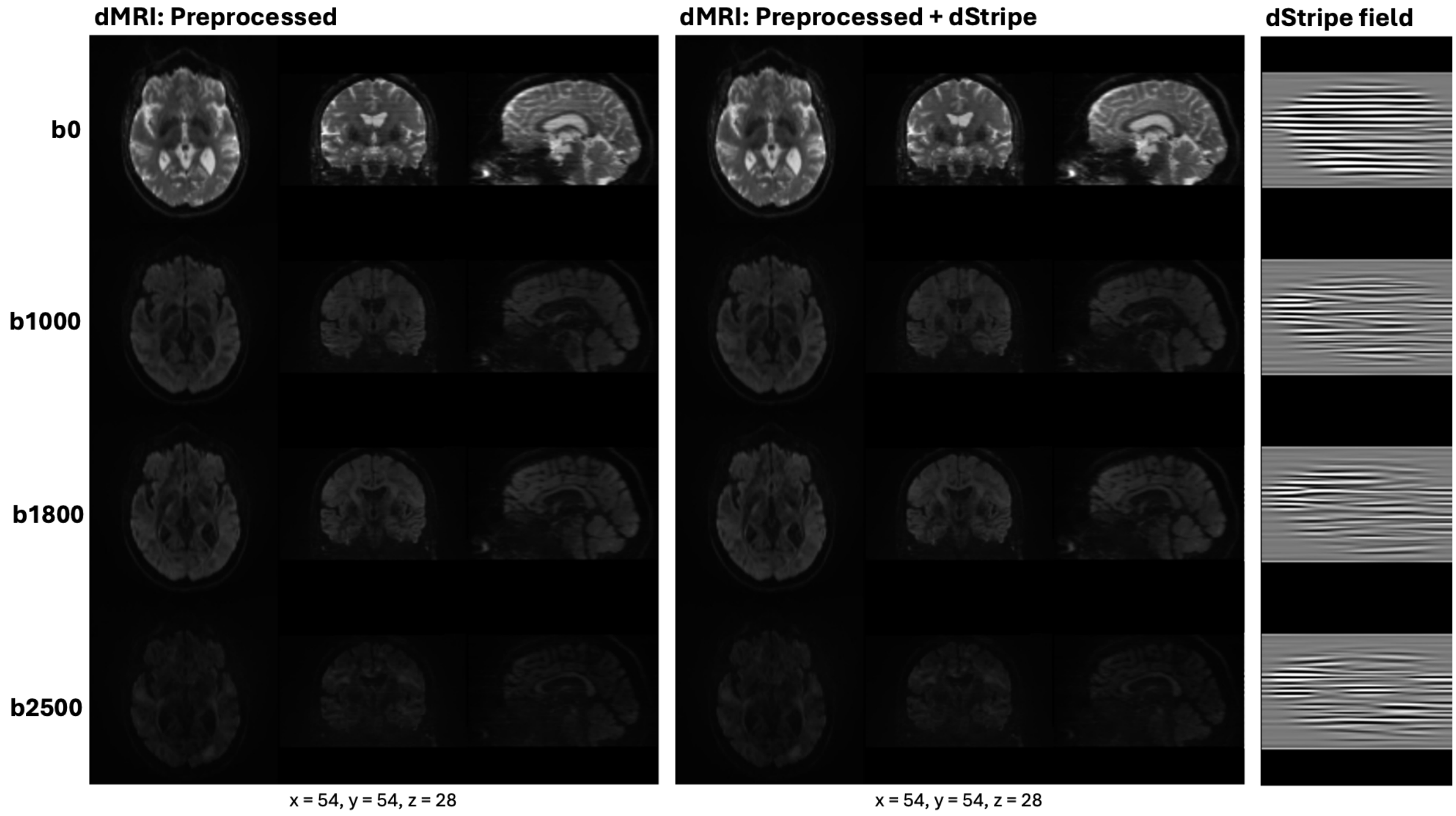

Figure S1: *Diffusion-weighted MRI (dMRI) of a representative participant (ID: PREDICT38355) with moderate stripe artefacts pre (left panel) and post (middle panel) ‚dStripe' processing. Stripe artefacts are visible in coronal and sagittal view. Right panel shows the multiplication field in sagittal view derived from the ‚dStripe' tool.*

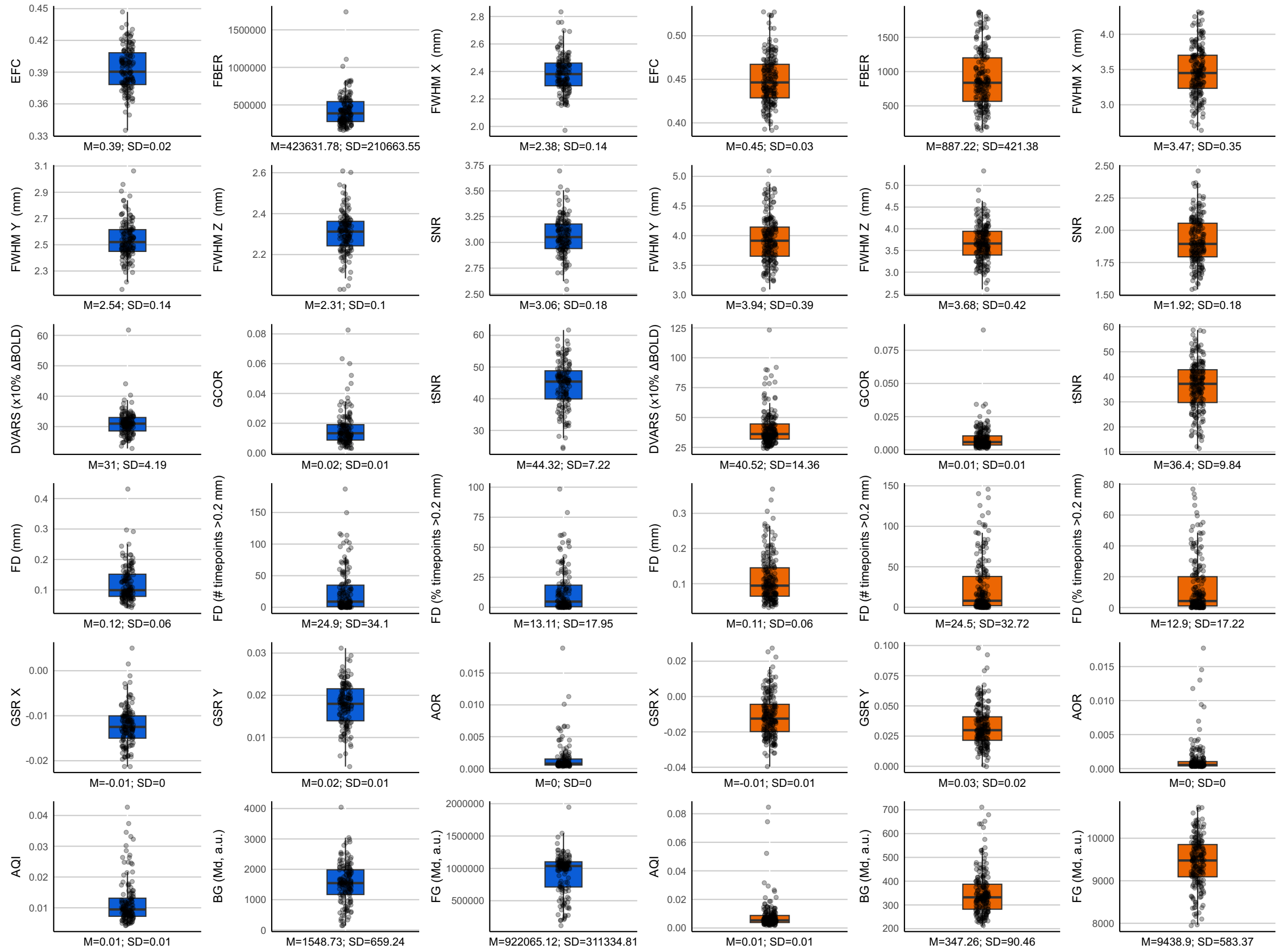

Figure S2: *Image quality metrics of fMRI data derived from MRIQC: Shown separately for included datasets acquired on the Philips Achieva 3T (blue) and Siemens Prisma 3T (orange). Measures relating to spatial information: Entropy-focus criterion (EFC), foreground-background energy ratio (FBER), full-width half maximum smoothness (FWHM), signal-to-noise ratio (SNR). Measures relating to temporal information: DVARS, global correlation (GCOR), temporal SNR (tSNR). Measures relating to artefacts and other: Framewise displacement (FD), ghost-to-signal ratio (GSR), AFNI's outlier ratio (AOR), AFNI's quality index (AQI). Summary statistics: Median background values (BG), median foreground values (FG). Beware that IQMs based on background intensities vary between scanners due to vendor-specific background intensities; the background in Philips DICOMs is set to zero. (Abbr.) M, mean; Md, median; SD, standard deviation.*

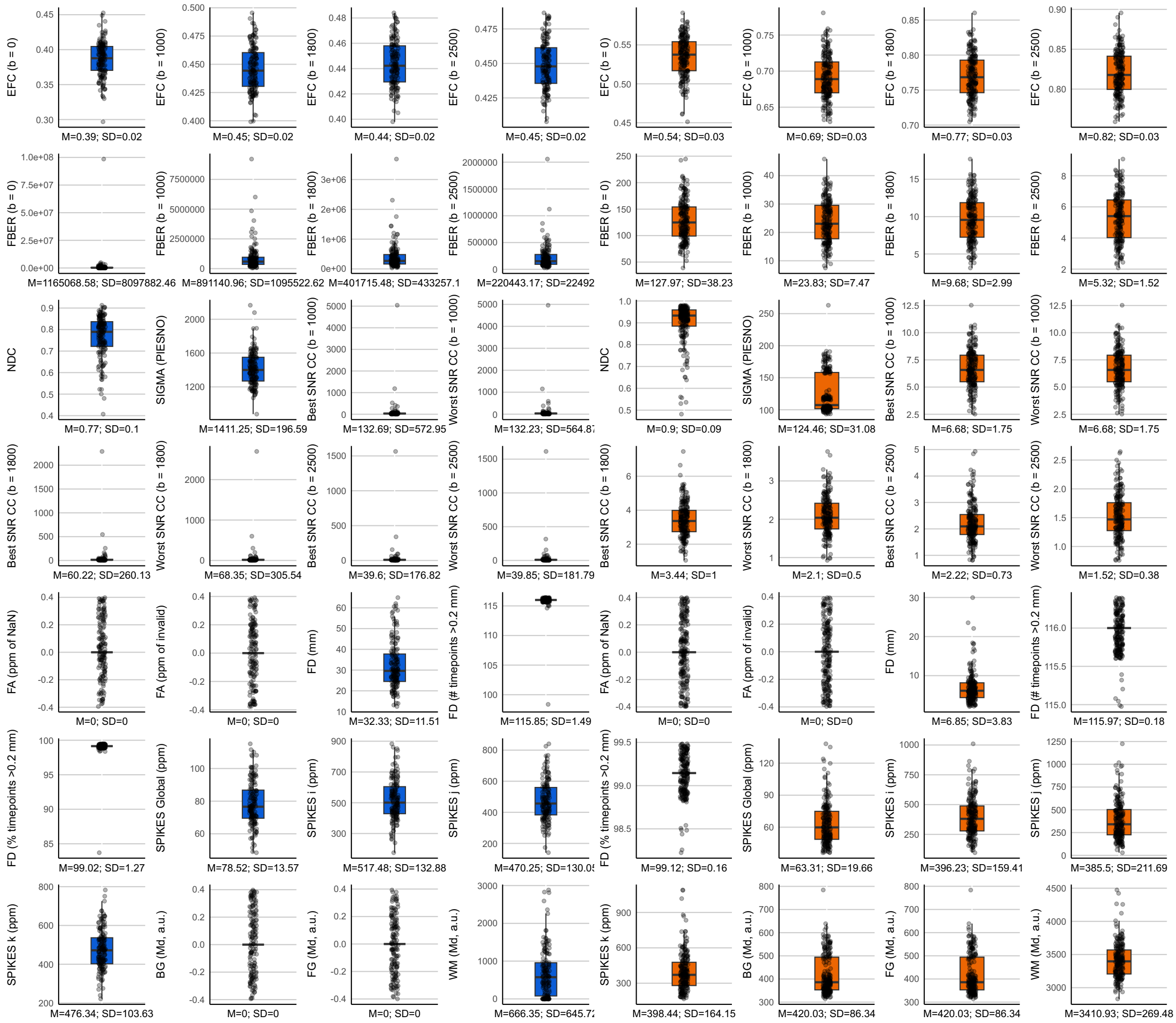

Figure S3: *Image quality metrics of dMRI data derived from MRIQC: Shown separately for included datasets acquired on the Philips Achieva 3T (blue) and Siemens Prisma 3T (orange). Measures relating to spatial information: Entropy-focus criterion (EFC), foreground-background energy ratio (FBER). Measures relating to diffusion weighting: Neighbouring DWI correlation (NDC), standard deviation of noise in raw dMRI estimated with PIESNO (SIGMA), Best-case and worst-case signal-to-noise ratio within corpus callosum (SNR CC), number of NaN values in FA map, number of invalid FA values within brain mask. Measures relating to artefacts and other: Framewise displacement (FD), global and slice-wise spike fractions (SPIKES). Summary statistics: Median background values (BG), median foreground values (FG), median white matter values (WM). Beware that IQMs based on background intensities vary between scanners due to vendor-specific background intensities; the background in Philips DICOMs is set to zero. (Abbr.) M, mean; Md, median; ppm, parts-per-million; SD, standard deviation.*

2 Methods – Connectome construction

Table S2: *Look-up table of the Brainnetome atlas, including classification into Yeo7's large-scale functional networks. For more information please visit:* http://atlas.brainnetome.org/bnatlas.html

	**Name**	**ROI**	**Modified cyto-architectonic**	**Yeo7**	**MNI (x; y; z)**		
1	Superior Frontal Gyrus	SFG_L_7_1	A8m_L	CEN	-5	15	54
2	Superior Frontal Gyrus	SFG_R_7_1	A8m_R	VAN	7	16	54
3	Superior Frontal Gyrus	SFG_L_7_2	A8dl_L	DMN	-18	24	53
4	Superior Frontal Gyrus	SFG_R_7_2	A8dl_R	CEN	22	26	51
5	Superior Frontal Gyrus	SFG_L_7_3	A9l_L	DMN	-11	49	40
6	Superior Frontal Gyrus	SFG_R_7_3	A9l_R	DMN	13	48	40
7	Superior Frontal Gyrus	SFG_L_7_4	A6dl_L	DAN	-18	-1	65
8	Superior Frontal Gyrus	SFG_R_7_4	A6dl_R	DAN	20	4	64
9	Superior Frontal Gyrus	SFG_L_7_5	A6m_L	SMN	-6	-5	58
10	Superior Frontal Gyrus	SFG_R_7_5	A6m_R	SMN	7	-4	60
11	Superior Frontal Gyrus	SFG_L_7_6	A9m_L	DMN	-5	36	38
12	Superior Frontal Gyrus	SFG_R_7_6	A9m_R	CEN	6	38	35
13	Superior Frontal Gyrus	SFG_L_7_7	A10m_L	DMN	-8	56	15
14	Superior Frontal Gyrus	SFG_R_7_7	A10m_R	DMN	8	58	13
15	Middle Frontal Gyrus	MFG_L_7_1	A9/46d_L	VAN	-27	43	31
16	Middle Frontal Gyrus	MFG_R_7_1	A9/46d_R	CEN	30	37	36
17	Middle Frontal Gyrus	MFG_L_7_2	IFJ_L	CEN	-42	13	36
18	Middle Frontal Gyrus	MFG_R_7_2	IFJ_R	CEN	42	11	39
19	Middle Frontal Gyrus	MFG_L_7_3	A46_L	CEN	-28	56	12
20	Middle Frontal Gyrus	MFG_R_7_3	A46_R	CEN	28	55	17
21	Middle Frontal Gyrus	MFG_L_7_4	A9/46v_L	CEN	-41	41	16
22	Middle Frontal Gyrus	MFG_R_7_4	A9/46v_R	CEN	42	44	14
23	Middle Frontal Gyrus	MFG_L_7_5	A8vl_L	DMN	-33	23	45
24	Middle Frontal Gyrus	MFG_R_7_5	A8vl_R	CEN	42	27	39
25	Middle Frontal Gyrus	MFG_L_7_6	A6vl_L	DAN	-32	4	55
26	Middle Frontal Gyrus	MFG_R_7_6	A6vl_R	DAN	34	8	54
27	Middle Frontal Gyrus	MFG_L_7_7	A10l_L	LIM	-26	60	-6
28	Middle Frontal Gyrus	MFG_R_7_7	A10l_R	CEN	25	61	-4
29	Inferior Frontal Gyrus	IFG_L_6_1	A44d_L	CEN	-46	13	24
30	Inferior Frontal Gyrus	IFG_R_6_1	A44d_R	DAN	45	16	25
31	Inferior Frontal Gyrus	IFG_L_6_2	IFS_L	CEN	-47	32	14
32	Inferior Frontal Gyrus	IFG_R_6_2	IFS_R	CEN	48	35	13
33	Inferior Frontal Gyrus	IFG_L_6_3	A45c_L	DMN	-53	23	11
34	Inferior Frontal Gyrus	IFG_R_6_3	A45c_R	DMN	54	24	12
35	Inferior Frontal Gyrus	IFG_L_6_4	A45r_L	DMN	-49	36	-3
36	Inferior Frontal Gyrus	IFG_R_6_4	A45r_R	CEN	51	36	-1
37	Inferior Frontal Gyrus	IFG_L_6_5	A44op_L	VAN	-39	23	4
38	Inferior Frontal Gyrus	IFG_R_6_5	A44op_R	VAN	42	22	3
39	Inferior Frontal Gyrus	IFG_L_6_6	A44v_L	VAN	-52	13	6
40	Inferior Frontal Gyrus	IFG_R_6_6	A44v_R	VAN	54	14	11
41	Orbital Gyrus	OrG_L_6_1	A14m_L	DMN	-7	54	-7
42	Orbital Gyrus	OrG_R_6_1	A14m_R	DMN	6	47	-7
43	Orbital Gyrus	OrG_L_6_2	A12/47o_L	DMN	-36	33	-16
44	Orbital Gyrus	OrG_R_6_2	A12/47o_R	DMN	40	39	-14
45	Orbital Gyrus	OrG_L_6_3	A11l_L	LIM	-23	38	-18
46	Orbital Gyrus	OrG_R_6_3	A11l_R	CEN	23	36	-18
47	Orbital Gyrus	OrG_L_6_4	A11m_L	LIM	-6	52	-19
48	Orbital Gyrus	OrG_R_6_4	A11m_R	LIM	6	57	-16
49	Orbital Gyrus	OrG_L_6_5	A13_L	LIM	-10	18	-19
50	Orbital Gyrus	OrG_R_6_5	A13_R	LIM	9	20	-19
51	Orbital Gyrus	OrG_L_6_6	A12/47l_L	DMN	-41	32	-9
52	Orbital Gyrus	OrG_R_6_6	A12/47l_R	DMN	42	31	-9
53	Precentral Gyrus	PrG_L_6_1	A4hf_L	SMN	-49	-8	39

54	Precentral Gyrus	PrG_R_6_1	A4hf_R	SMN	55	-2	33
55	Precentral Gyrus	PrG_L_6_2	A6cdl_L	DAN	-32	-9	58
56	Precentral Gyrus	PrG_R_6_2	A6cdl_R	DAN	33	-7	57
57	Precentral Gyrus	PrG_L_6_3	A4ul_L	SMN	-26	-25	63
58	Precentral Gyrus	PrG_R_6_3	A4ul_R	SMN	34	-19	59
59	Precentral Gyrus	PrG_L_6_4	A4t_L	SMN	-13	-20	73
60	Precentral Gyrus	PrG_R_6_4	A4t_R	SMN	15	-22	71
61	Precentral Gyrus	PrG_L_6_5	A4tl_L	VAN	-52	0	8
62	Precentral Gyrus	PrG_R_6_5	A4tl_R	VAN	54	4	9
63	Precentral Gyrus	PrG_L_6_6	A6cvl_L	DAN	-49	5	30
64	Precentral Gyrus	PrG_R_6_6	A6cvl_R	DAN	51	7	30
65	Paracentral Gyrus	PCL_L_2_1	A1/2/3ll_L	VAN	-8	-38	58
66	Paracentral Gyrus	PCL_R_2_1	A1/2/3ll_R	SMN	10	-34	54
67	Paracentral Gyrus	PCL_L_2_2	A4ll_L	SMN	-4	-23	61
68	Paracentral Gyrus	PCL_R_2_2	A4ll_R	SMN	5	-21	61
69	Superior Temporal Gyrus	STG_L_6_1	A38m_L	LIM	-32	14	-34
70	Superior Temporal Gyrus	STG_R_6_1	A38m_R	LIM	31	15	-34
71	Superior Temporal Gyrus	STG_L_6_2	A41/42_L	SMN	-54	-32	12
72	Superior Temporal Gyrus	STG_R_6_2	A41/42_R	SMN	54	-24	11
73	Superior Temporal Gyrus	STG_L_6_3	TE1.0/TE1.2_L	SMN	-50	-11	1
74	Superior Temporal Gyrus	STG_R_6_3	TE1.0/TE1.2_R	SMN	51	-4	-1
75	Superior Temporal Gyrus	STG_L_6_4	A22c_L	SMN	-62	-33	7
76	Superior Temporal Gyrus	STG_R_6_4	A22c_R	SMN	66	-20	6
77	Superior Temporal Gyrus	STG_L_6_5	A38l_L	LIM	-45	11	-20
78	Superior Temporal Gyrus	STG_R_6_5	A38l_R	LIM	47	12	-20
79	Superior Temporal Gyrus	STG_L_6_6	A22r_L	DMN	-55	-3	-10
80	Superior Temporal Gyrus	STG_R_6_6	A22r_R	DMN	56	-12	-5
81	Middle Temporal Gyrus	MTG_L_4_1	A21c_L	DMN	-65	-30	-12
82	Middle Temporal Gyrus	MTG_R_4_1	A21c_R	CEN	65	-29	-13
83	Middle Temporal Gyrus	MTG_L_4_2	A21r_L	DMN	-53	2	-30
84	Middle Temporal Gyrus	MTG_R_4_2	A21r_R	DMN	51	6	-32
85	Middle Temporal Gyrus	MTG_L_4_3	A37dl_L	DAN	-59	-58	4
86	Middle Temporal Gyrus	MTG_R_4_3	A37dl_R	DAN	60	-53	3
87	Middle Temporal Gyrus	MTG_L_4_4	aSTS_L	DMN	-58	-20	-9
88	Middle Temporal Gyrus	MTG_R_4_4	aSTS_R	DMN	58	-16	-10
89	Inferior Temporal Gyrus	ITG_L_7_1	A20iv_L	LIM	-45	-26	-27
90	Inferior Temporal Gyrus	ITG_R_7_1	A20iv_R	LIM	46	-14	-33
91	Inferior Temporal Gyrus	ITG_L_7_2	A37elv_L	DAN	-51	-57	-15
92	Inferior Temporal Gyrus	ITG_R_7_2	A37elv_R	DAN	53	-52	-18
93	Inferior Temporal Gyrus	ITG_L_7_3	A20r_L	LIM	-43	-2	-41
94	Inferior Temporal Gyrus	ITG_R_7_3	A20r_R	LIM	40	0	-43
95	Inferior Temporal Gyrus	ITG_L_7_4	A20il_L	DMN	-56	-16	-28
96	Inferior Temporal Gyrus	ITG_R_7_4	A20il_R	LIM	55	-11	-32
97	Inferior Temporal Gyrus	ITG_L_7_5	A37vl_L	DAN	-55	-60	-6
98	Inferior Temporal Gyrus	ITG_R_7_5	A37vl_R	DAN	54	-57	-8
99	Inferior Temporal Gyrus	ITG_L_7_6	A20cl_L	CEN	-59	-42	-16
100	Inferior Temporal Gyrus	ITG_R_7_6	A20cl_R	CEN	61	-40	-17
101	Inferior Temporal Gyrus	ITG_L_7_7	A20cv_L	LIM	-55	-31	-27
102	Inferior Temporal Gyrus	ITG_R_7_7	A20cv_R	LIM	54	-31	-26
103	Fusiform Gyrus	FuG_L_3_1	A20rv_L	LIM	-33	-16	-32
104	Fusiform Gyrus	FuG_R_3_1	A20rv_R	LIM	33	-15	-34
105	Fusiform Gyrus	FuG_L_3_2	A37mv_L	VIS	-31	-64	-14
106	Fusiform Gyrus	FuG_R_3_2	A37mv_R	VIS	31	-62	-14
107	Fusiform Gyrus	FuG_L_3_3	A37lv_L	DAN	-42	-51	-17
108	Fusiform Gyrus	FuG_R_3_3	A37lv_R	VIS	43	-49	-19
109	Parahippocampal Gyrus	PhG_L_6_1	A35/36r_L	LIM	-27	-7	-34
110	Parahippocampal Gyrus	PhG_R_6_1	A35/36r_R	LIM	28	-8	-33
111	Parahippocampal Gyrus	PhG_L_6_2	A35/36c_L	LIM	-25	-25	-26
112	Parahippocampal Gyrus	PhG_R_6_2	A35/36c_R	VIS	26	-23	-27
113	Parahippocampal Gyrus	PhG_L_6_3	TL_L	VIS	-28	-32	-18
114	Parahippocampal Gyrus	PhG_R_6_3	TL_R	VIS	30	-30	-18

115	Parahippocampal Gyrus	PhG_L_6_4	A28/34_L	LIM	-19	-12	-30
116	Parahippocampal Gyrus	PhG_R_6_4	A28/34_R	LIM	19	-10	-30
117	Parahippocampal Gyrus	PhG_L_6_5	TI_L	LIM	-23	2	-32
118	Parahippocampal Gyrus	PhG_R_6_5	TI_R	LIM	22	1	-36
119	Parahippocampal Gyrus	PhG_L_6_6	TH_L	VIS	-17	-39	-10
120	Parahippocampal Gyrus	PhG_R_6_6	TH_R	VIS	19	-36	-11
121	Posterior Superior Temporal Sulcus	pSTS_L_2_1	rpSTS_L	DMN	-54	-40	4
122	Posterior Superior Temporal Sulcus	pSTS_R_2_1	rpSTS_R	DMN	53	-37	3
123	Posterior Superior Temporal Sulcus	pSTS_L_2_2	cpSTS_L	VAN	-52	-50	11
124	Posterior Superior Temporal Sulcus	pSTS_R_2_2	cpSTS_R	VAN	57	-40	12
125	Superior Parietal Lobule	SPL_L_5_1	A7r_L	DAN	-16	-60	63
126	Superior Parietal Lobule	SPL_R_5_1	A7r_R	DAN	19	-57	65
127	Superior Parietal Lobule	SPL_L_5_2	A7c_L	DAN	-15	-71	52
128	Superior Parietal Lobule	SPL_R_5_2	A7c_R	DAN	19	-69	54
129	Superior Parietal Lobule	SPL_L_5_3	A5l_L	DAN	-33	-47	50
130	Superior Parietal Lobule	SPL_R_5_3	A5l_R	DAN	35	-42	54
131	Superior Parietal Lobule	SPL_L_5_4	A7pc_L	SMN	-22	-47	65
132	Superior Parietal Lobule	SPL_R_5_4	A7pc_R	SMN	23	-43	67
133	Superior Parietal Lobule	SPL_L_5_5	A7ip_L	DAN	-27	-59	54
134	Superior Parietal Lobule	SPL_R_5_5	A7ip_R	DAN	31	-54	53
135	Inferior Parietal Lobule	IPL_L_6_1	A39c_L	VIS	-34	-80	29
136	Inferior Parietal Lobule	IPL_R_6_1	A39c_R	VIS	45	-71	20
137	Inferior Parietal Lobule	IPL_L_6_2	A39rd_L	CEN	-38	-61	46
138	Inferior Parietal Lobule	IPL_R_6_2	A39rd_R	CEN	39	-65	44
139	Inferior Parietal Lobule	IPL_L_6_3	A40rd_L	DAN	-51	-33	42
140	Inferior Parietal Lobule	IPL_R_6_3	A40rd_R	DAN	47	-35	45
141	Inferior Parietal Lobule	IPL_L_6_4	A40c_L	DMN	-56	-49	38
142	Inferior Parietal Lobule	IPL_R_6_4	A40c_R	CEN	57	-44	38
143	Inferior Parietal Lobule	IPL_L_6_5	A39rv_L	DAN	-47	-65	26
144	Inferior Parietal Lobule	IPL_R_6_5	A39rv_R	DMN	53	-54	25
145	Inferior Parietal Lobule	IPL_L_6_6	A40rv_L	SMN	-53	-31	23
146	Inferior Parietal Lobule	IPL_R_6_6	A40rv_R	SMN	55	-26	26
147	Precuneus	PCun_L_4_1	A7m_L	CEN	-5	-63	51
148	Precuneus	PCun_R_4_1	A7m_R	CEN	6	-65	51
149	Precuneus	PCun_L_4_2	A5m_L	SMN	-8	-47	57
150	Precuneus	PCun_R_4_2	A5m_R	DAN	7	-47	58
151	Precuneus	PCun_L_4_3	dmPOS_L	VIS	-12	-67	25
152	Precuneus	PCun_R_4_3	dmPOS_R	VIS	16	-64	25
153	Precuneus	PCun_L_4_4	A31_L	DMN	-6	-55	34
154	Precuneus	PCun_R_4_4	A31_R	DMN	6	-54	35
155	Postcentral Gyrus	PoG_L_4_1	A1/2/3ulhf_L	SMN	-50	-16	43
156	Postcentral Gyrus	PoG_R_4_1	A1/2/3ulhf_R	SMN	50	-14	44
157	Postcentral Gyrus	PoG_L_4_2	A1/2/3tonIa_L	SMN	-56	-14	16
158	Postcentral Gyrus	PoG_R_4_2	A1/2/3tonIa_R	SMN	56	-10	15
159	Postcentral Gyrus	PoG_L_4_3	A2_L	DAN	-46	-30	50
160	Postcentral Gyrus	PoG_R_4_3	A2_R	SMN	48	-24	48
161	Postcentral Gyrus	PoG_L_4_4	A1/2/3tru_L	SMN	-21	-35	68
162	Postcentral Gyrus	PoG_R_4_4	A1/2/3tru_R	SMN	20	-33	69
163	Insular Gyrus	INS_L_6_1	G_L	SMN	-36	-20	10
164	Insular Gyrus	INS_R_6_1	G_R	SMN	37	-18	8
165	Insular Gyrus	INS_L_6_2	vIa_L	SUBC	-32	14	-13
166	Insular Gyrus	INS_R_6_2	vIa_R	CEN	33	14	-13
167	Insular Gyrus	INS_L_6_3	dIa_L	VAN	-34	18	1
168	Insular Gyrus	INS_R_6_3	dIa_R	VAN	36	18	1
169	Insular Gyrus	INS_L_6_4	vId/vIg_L	VAN	-38	-4	-9
170	Insular Gyrus	INS_R_6_4	vId/vIg_R	VAN	39	-2	-9
171	Insular Gyrus	INS_L_6_5	dIg_L	SMN	-38	-8	8

172	Insular Gyrus	INS_R_6_5	dIg_R	SMN	39	-7	8
173	Insular Gyrus	INS_L_6_6	dId_L	VAN	-38	5	5
174	Insular Gyrus	INS_R_6_6	dId_R	VAN	38	5	5
175	Cingulate Gyrus	CG_L_7_1	A23d_L	DMN	-4	-39	31
176	Cingulate Gyrus	CG_R_7_1	A23d_R	DMN	4	-37	32
177	Cingulate Gyrus	CG_L_7_2	A24rv_L	SUBC	-3	8	25
178	Cingulate Gyrus	CG_R_7_2	A24rv_R	SUBC	5	22	12
179	Cingulate Gyrus	CG_L_7_3	A32p_L	DMN	-6	34	21
180	Cingulate Gyrus	CG_R_7_3	A32p_R	VAN	5	28	27
181	Cingulate Gyrus	CG_L_7_4	A23v_L	DMN	-8	-47	10
182	Cingulate Gyrus	CG_R_7_4	A23v_R	VIS	9	-44	11
183	Cingulate Gyrus	CG_L_7_5	A24cd_L	VAN	-5	7	37
184	Cingulate Gyrus	CG_R_7_5	A24cd_R	VAN	4	6	38
185	Cingulate Gyrus	CG_L_7_6	A23c_L	VAN	-7	-23	41
186	Cingulate Gyrus	CG_R_7_6	A23c_R	VAN	6	-20	40
187	Cingulate Gyrus	CG_L_7_7	A32sg_L	DMN	-4	39	-2
188	Cingulate Gyrus	CG_R_7_7	A32sg_R	DMN	5	41	6
189	Medioventral Occipital Cortex	MVOcC_L_5_1	cLinG_L	VIS	-11	-82	-11
190	Medioventral Occipital Cortex	MVOcC_R_5_1	cLinG_R	VIS	10	-85	-9
191	Medioventral Occipital Cortex	MVOcC_L_5_2	rCunG_L	VIS	-5	-81	10
192	Medioventral Occipital Cortex	MVOcC_R_5_2	rCunG_R	VIS	7	-76	11
193	Medioventral Occipital Cortex	MVOcC_L_5_3	cCunG_L	VIS	-6	-94	1
194	Medioventral Occipital Cortex	MVOcC_R_5_3	cCunG_R	VIS	8	-90	12
195	Medioventral Occipital Cortex	MVOcC_L_5_4	rLinG_L	VIS	-17	-60	-6
196	Medioventral Occipital Cortex	MVOcC_R_5_4	rLinG_R	VIS	18	-60	-7
197	Medioventral Occipital Cortex	MVOcC_L_5_5	vmPOS_L	VIS	-13	-68	12
198	Medioventral Occipital Cortex	MVOcC_R_5_5	vmPOS_R	VIS	15	-63	12
199	Lateral Occipital Cortex	LOcC_L_4_1	mOccG_L	VIS	-31	-89	11
200	Lateral Occipital Cortex	LOcC_R_4_1	mOccG_R	VIS	34	-86	11
201	Lateral Occipital Cortex	LOcC_L_4_2	V5/MT+_L	DAN	-46	-74	3
202	Lateral Occipital Cortex	LOcC_R_4_2	V5/MT+_R	VIS	48	-70	-1
203	Lateral Occipital Cortex	LOcC_L_4_3	OPC_L	VIS	-18	-99	2
204	Lateral Occipital Cortex	LOcC_R_4_3	OPC_R	VIS	22	-97	4
205	Lateral Occipital Cortex	LOcC_L_4_4	iOccG_L	VIS	-30	-88	-12
206	Lateral Occipital Cortex	LOcC_R_4_4	iOccG_R	VIS	32	-85	-12
207	Lateral Occipital Cortex	LOcC_L_2_1	msOccG_L	VIS	-11	-88	31
208	Lateral Occipital Cortex	LOcC_R_2_1	msOccG_R	VIS	16	-85	34
209	Lateral Occipital Cortex	LOcC_L_2_2	lsOccG_L	VIS	-22	-77	36
210	Lateral Occipital Cortex	LOcC_R_2_2	lsOccG_R	VIS	29	-75	36
211	Amygdala	Amyg_L_2_1	mAmyg_L	SUBC	-19	-2	-20
212	Amygdala	Amyg_R_2_1	mAmyg_R	SUBC	19	-2	-19
213	Amygdala	Amyg_L_2_2	lAmyg_L	SUBC	-27	-4	-20
214	Amygdala	Amyg_R_2_2	lAmyg_R	SUBC	28	-3	-20
215	Hippocampus	Hipp_L_2_1	rHipp_L	SUBC	-22	-14	-19
216	Hippocampus	Hipp_R_2_1	rHipp_R	SUBC	22	-12	-20
217	Hippocampus	Hipp_L_2_2	cHipp_L	SUBC	-28	-30	-10
218	Hippocampus	Hipp_R_2_2	cHipp_R	SUBC	29	-27	-10
219	Caudate	BG_L_6_1	vCa_L	SUBC	-12	14	0
220	Caudate	BG_R_6_1	vCa_R	SUBC	15	14	-2
221	Globus Pallidus	BG_L_6_2	GP_L	SUBC	-22	-2	4
222	Globus Pallidus	BG_R_6_2	GP_R	SUBC	22	-2	3
223	Nucleus Accumbens	BG_L_6_3	NAC_L	SUBC	-17	3	-9
224	Nucleus Accumbens	BG_R_6_3	NAC_R	SUBC	15	8	-9
225	Putamen	BG_L_6_4	vmPu_L	SUBC	-23	7	-4
226	Putamen	BG_R_6_4	vmPu_R	SUBC	22	8	-1
227	Caudate	BG_L_6_5	dCa_L	SUBC	-14	2	16
228	Caudate	BG_R_6_5	dCa_R	SUBC	14	5	14
229	Putamen	BG_L_6_6	dlPu_L	SUBC	-28	-5	2
230	Putamen	BG_R_6_6	dlPu_R	SUBC	29	-3	1
231	Thalmus	Tha_L_8_1	mPFtha_L	SUBC	-7	-12	5
232	Thalmus	Tha_R_8_1	mPFtha_R	SUBC	7	-11	6

233	Thalmus	Tha_L_8_2	mPMtha_L	SUBC	-18	-13	3
234	Thalmus	Tha_R_8_2	mPMtha_R	SUBC	12	-14	1
235	Thalmus	Tha_L_8_3	Stha_L	SUBC	-18	-23	4
236	Thalmus	Tha_R_8_3	Stha_R	SUBC	18	-22	3
237	Thalmus	Tha_L_8_4	rTtha_L	SUBC	-7	-14	7
238	Thalmus	Tha_R_8_4	rTtha_R	SUBC	3	-13	5
239	Thalmus	Tha_L_8_5	PPtha_L	SUBC	-16	-24	6
240	Thalmus	Tha_R_8_5	PPtha_R	SUBC	15	-25	6
241	Thalmus	Tha_L_8_6	Otha_L	SUBC	-15	-28	4
242	Thalmus	Tha_R_8_6	Otha_R	SUBC	13	-27	8
243	Thalmus	Tha_L_8_7	cTtha_L	SUBC	-12	-22	13
244	Thalmus	Tha_R_8_7	cTtha_R	SUBC	10	-14	14
245	Thalmus	Tha_L_8_8	lPFtha_L	SUBC	-11	-14	2
246	Thalmus	Tha_R_8_8	lPFtha_R	SUBC	13	-16	7

3 Methods – Structure-function coupling

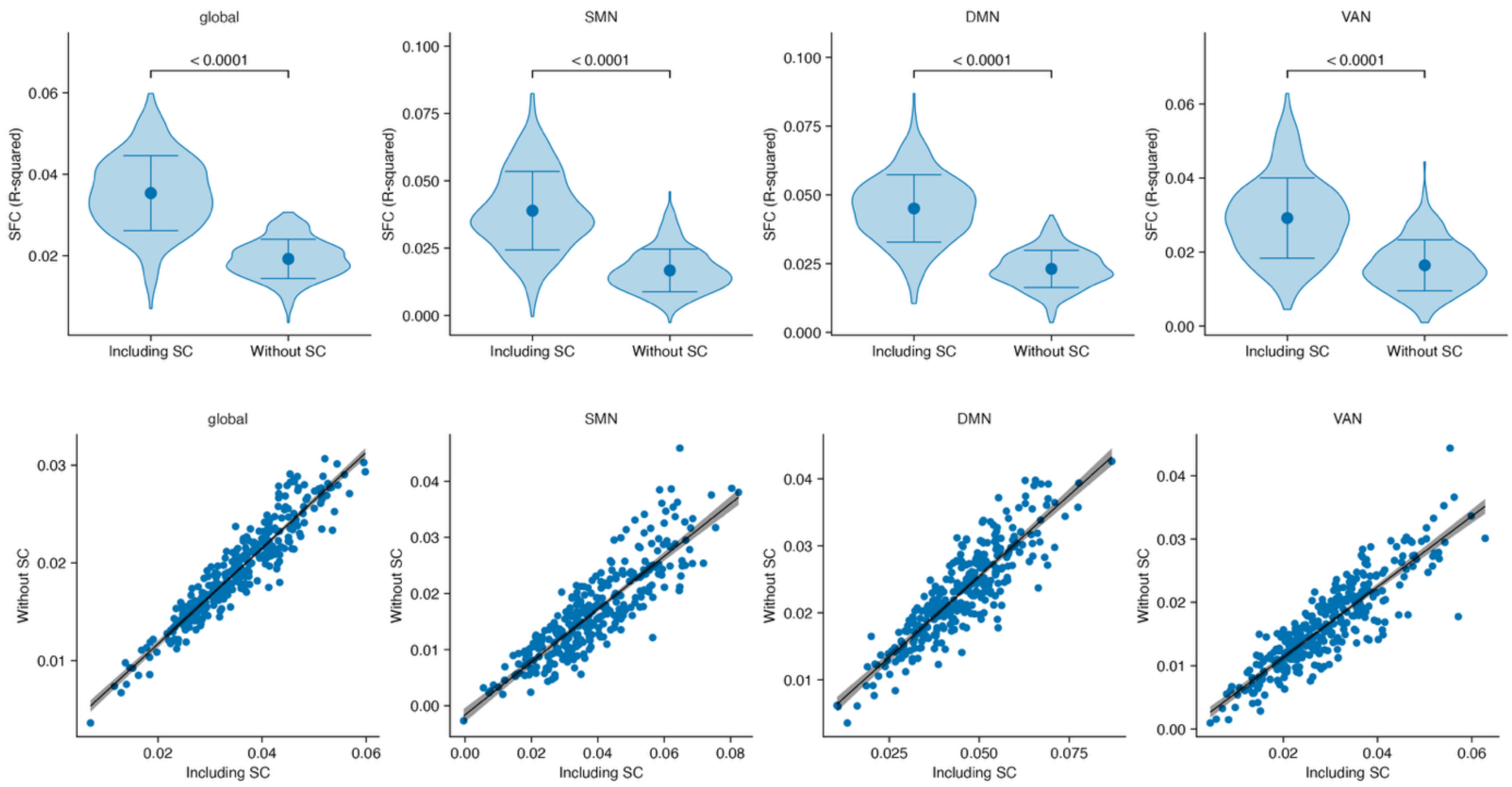

Figure S4: *Comparison between two multivariable models of structure-function coupling (SFC). ‚Including SC‘ represents the modelt hat was used in the analyses of the paper and integrates four predictors: : structural connectivity (SC), shortest path length, communicability, and Euclidean distance. ‘Without SC’ is the model reduced to three communication predictors and is commonly used in model-based SFC approaches. Here, we demonstrate that by including SC as a predictor, the explained variance (R2 = SFC) of the model increased significantly while both approaches show a high Pearson corelation coefficient (global: r = 0.94, SMN: r = 0.86, DMN: r = 0.87, VAN r = 0.88; all p < 0.001).*

Table S3: *The variance inflation factor (VIF) compared between the two multivariable models of structrue-function coupling. All predictors have an VIF < 5, suggesting non-collinearity.*

	**Variance inflation factor**	
	Three-predictor model	Four-predictor model
**SC**	-	1.24 ± 0.89
**PL**	2.02 ± 0.63	2.12 ± 0.61
**COM**	1.02 ± 0.02	1.02 ± 0.02
**ED**	2.02 ± 0.64	2.07 ± 0.63

Values in M ± SD across all participants and nodes.

4 Results – Participants

Table S4: *Distribution of patients with LBP and pain-free controls across the two scanners and the dMRI protocols.*

Group	Scanner (PHIL; SIEM)	dMRI protocol (PHIL; SIEM-1; SIEM-2)
Main groups:		
CG	38; 33 (54%; 46%)	38; 13; 19 (54%; 19%; 27%)
LBP–	32; 36 (47%; 53%)	32; 30; 6 (47%; 44%; 9%)
LBP+	78; 136 (36%; 64%)	78; 104; 31 (36%; 49%; 15%)
Subgroups:		
cLBP	44; 78 (36%; 64%)	44 / 59 / 18 (36%; 49%; 15%)
ncLBP	16; 31 (34%; 66%)	16 / 25 / 6 (34%; 53%; 13%)

Values in *n* (%)

5 Results – Structural and functional topology

Table S5: *Group comparisons of structural global network properties for the full dataset.*

Global metric	LBP+ (n = 197)	LBP– (n = 66)	CG (n = 63)	Global test (p_{FDR})	Post-hoc test (p)
**Whole-brain structural connectome**					
CC	0.918 ± 0.016	0.918 ± 0.011	0.916 ± 0.016	0.760	–
E_{glob}	410.1 ± 26.1	407.5 ± 24.4	411.1 ± 20.9	0. 760	–
E_{loc}	415.4 ± 26.9	412.9 ± 25.5	417.1 ± 21.2	0. 760	–
L (×10^3)	3.184 ± 0.25	3.203 ± 0.24	3.174 ± 0.203	0. 760	–
Q	0.570 ± 0.013	0.571 ± 0.013	0.572 ± 0.011	0. 760	–
**Structural default mode network (sDMN)**					
CC	0.942 ± 0.018	0.941 ± 0.018	0.935 ± 0.018	0.182	–
E_{glob}	299.3 ± 27	294.2 ± 30.7	297.8 ± 24.9	0.368	–
E_{loc}	304.1 ± 27.6	299.3 ± 31.2	304.5 ± 23.8	0.368	–
L (×10^3)	13.20 ± 3.06	13.69 ± 3.74	14.44 ± 4.08	0.182	–
Q	0.674 ± 0.019	0.676 ± 0.02	0.682 ± 0.019	0.078	–
**Structural somatomotor network (sSMN)**					
CC	0.974 ± 0.023	0.973 ± 0.022	0.97 ± 0.019	0.988	–
E_{glob}	600.6 ± 67.4	605 ± 73.9	605.6 ± 62.4	0.988	–
E_{loc}	593.4 ± 66.3	598.7 ± 72.9	600.7 ± 62.5	0.988	–
L (×10^3)	3.28 ± 0.538	3.267 ± 0.624	3.218 ± 0.435	0.988	–
Q	0.577 ± 0.026	0.581 ± 0.03	0.579 ± 0.022	0.988	–
**Structural ventral attention network (sVAN)**					
CC	0.95 ± 0.032	0.943 ± 0.044	0.933 ± 0.035	0.820	–
E_{glob}	192.1 ± 23.2	190.3 ± 22.1	192.8 ± 23.4	0.820	–
E_{loc}	194 ± 24	192.7 ± 23.5	196.5 ± 23.4	0.820	–
L (×10^3)	17.54 ± 4.51	17.10 ± 4.65	17.08 ± 4.57	0.820	–
Q	0.614 ± 0.029	0.616 ± 0.029	0.616 ± 0.02	0.820	–

Note. Mean ± SD, not adjusted for age, sex, and scanner. Bold text indicates significant results. CC, clustering coefficient; CG, control group; E_{glob}, global efficiency; E_{loc}, local efficiency; L, characteristic path length; LBP+, low back pain with pain during scanning; LBP–, low back pain without pain during scanning; Q, modularity.

Table S6*: Group comparisons of functional global network properties for the full dataset.*

**Global metric**	**LBP+ (n = 211)**	**LBP– (n = 68)**	**CG (n = 70)**	**Global test (p_{FDR})**	**Post-hoc test (p)**
**Whole-brain functional connectome**					
CC $(\times 10^3)$	8.809 ± 0.389	8.763 ± 0.382	8.946 ± 0.437	0.162	–
E_{glob} $(\times 10^3)$	1.256 ± 0.046	1.251 ± 0.057	1.257 ± 0.059	0.512	–
E_{loc} $(\times 10^3)$	1.33 ± 0.066	1.321 ± 0.075	1.339 ± 0.081	0.512	–
L	7.982 ± 0.605	7.983 ± 0.642	7.912 ± 0.676	0.796	–
Q $(\times 10^3)$	3.265 ± 0.131	3.301 ± 0.137	3.337 ± 0.173	0.088	–
**Functional default mode network (fDMN)**					
CC $(\times 10^3)$	9.19 ± 0.813	9.324 ± 0.832	9.524 ± 0.844	0.133	–
E_{glob} $(\times 10^3)$	1.063 ± 0.077	1.05 ± 0.072	1.053 ± 0.078	0.133	–
E_{loc} $(\times 10^3)$	1.11 ± 0.088	1.101 ± 0.088	1.114 ± 0.092	0.484	–
L	9.887 ± 0.747	9.984 ± 0.703	10.023 ± 0.805	0.133	–
Q $(\times 10^3)$	8.775 ± 0.688	8.962 ± 0.685	9.088 ± 0.736	0.110	–
**Functional somatomotor network (fSMN)**					
CC $(\times 10^3)$	9.64 ± 1.147	9.644 ± 1.384	10.17 ± 1.411	0.179	–
E_{glob} $(\times 10^3)$	1.156 ± 0.091	1.136 ± 0.087	1.161 ± 0.09	0.201	–
E_{loc} $(\times 10^3)$	1.224 ± 0.106	1.205 ± 0.105	1.25 ± 0.108	0.201	–
L	9.212 ± 0.775	9.389 ± 0.772	9.197 ± 0.755	0.201	–
Q $(\times 10^3)$	9.569 ± 0.928	9.48 ± 1.15	9.858 ± 1.157	0.201	–
**Functional ventral attention network (fVAN)**					
CC $(\times 10^3)$	8.415 ± 1.263	8.324 ± 1.231	8.829 ± 1.305	0.091	–
E_{glob} $(\times 10^3)$	1.105 ± 0.086	1.09 ± 0.085	1.088 ± 0.09	**0.043**	**CG vs LBP+ (.005)**<br>CG vs LBP– (.286)<br>LBP– vs LBP+ (.093)
E_{loc} $(\times 10^3)$	1.037 ± 0.09	1.024 ± 0.1	1.032 ± 0.097	0.177	–
L	10.043 ± 0.788	10.181 ± 0.87	10.268 ± 0.937	**0.043**	**CG vs LBP+ (.013)**<br>CG vs LBP– (.449)<br>LBP– vs LBP+ (.068)
Q $(\times 10^3)$	9.36 ± 0.666	9.335 ± 0.595	9.479 ± 0.748	0.452	–

Note. Mean ± SD, not adjusted for age, sex, and scanner. Bold text indicates significant results. CC, clustering coefficient; CG, pain-free control group; E_{glob}, global efficiency; E_{loc}, local efficiency; L, characteristic path length; LBP+, low back pain with pain during scanning; LBP–, low back pain without pain during scanning; Q, modularity.

Table S7: *Group comparisons of structural global network properties for the chronic and non-chronic LBP subsample of the LBP+ group.*

**Global metric**	**cLBP (n = 113)**	**ncLBP (n = 42)**	**CG (n = 63)**	**Global test (p_{FDR})**	**Post-hoc test (p)**
**Whole-brain structural connectome**					
CC	0.917 ± 0.017	0.918 ± 0.013	0.916 ± 0.016	0.677	–
E_{glob}	409.8 ± 25.7	405.7 ± 31.4	411.1 ± 20.9	0.677	–
E_{loc}	415 ± 26.4	410.6 ± 32.3	417.1 ± 21.2	0.677	–

L (×10³)	3.185 ± 0.244	3.229 ± 0.319	3.174 ± 0.203	0.677	–
Q	0.570 ± 0.012	0.571 ± 0.013	0.572 ± 0.011	0.677	–
**Structural default mode network (sDMN)**					
CC	0.943 ± 0.018	0.942 ± 0.018	0.935 ± 0.018	0.082	–
E_{glob}	301.3 ± 25.4	291.6 ± 32.8	297.8 ± 24.9	0.123	–
E_{loc}	305.8 ± 26.4	296.6 ± 33.6	304.5 ± 23.8	0.163	–
L (×10³)	12.94 ± 2.92	13.9 ± 2.89	14.44 ± 4.08	**0.024**	CG vs ncLBP (.534)<br>**CG vs cLBP (.003)**<br>**ncLBP vs cLBP (.027)**
Q	0.674 ± 0.019	0.673 ± 0.019	0.682 ± 0.019	**0.024**	CG vs ncLBP (.104)<br>**CG vs cLBP (.002)**<br>ncLBP vs cLBP (.773)
**Structural somatomotor network (sSMN)**					
CC	0.972 ± 0.025	0.975 ± 0.020	0.97 ± 0.019	0.841	–
E_{glob}	602 ± 73.3	582.1 ± 59.0	605.6 ± 62.4	0.285	–
E_{loc}	594.9 ± 71.7	574.8 ± 58.8	600.7 ± 62.5	0.285	–
L (×10³)	3.278 ± 0.545	3.449 ± 0.592	3.218 ± 0.435	0.285	–
Q	0.578 ± 0.026	0.579 ± 0.025	0.579 ± 0.022	0.841	–
**Structural ventral attention network (sVAN)**					
CC	0.95 ± 0.033	0.953 ± 0.027	0.933 ± 0.035	0.758	–
E_{glob}	191.6 ± 22.6	188.4 ± 24.1	192.8 ± 23.4	0.758	–
E_{loc}	193.4 ± 23.3	189.1 ± 25.4	196.5 ± 23.4	0.758	–
L (×10³)	17.62 ± 4.48	18.06 ± 4.85	17.08 ± 4.57	0.758	–
Q	0.612 ± 0.03	0.623 ± 0.023	0.616 ± 0.02	0.343	–

Note. Mean ± SD, not adjusted for age, sex, and scanner. Bold text indicates significant results. CC, clustering coefficient; CG, pain-free control group; E_{glob}, global efficiency; E_{loc}, local efficiency; L, characteristic path length; LBP+, low back pain with pain during scanning; LBP–, low back pain without pain during scanning; Q, modularity.

Table S8*: Group comparisons of functional global network properties for the chronic and non-chronic subsample of the LBP+ group.*

Global metric	cLBP (n = 120)	ncLBP (n = 47)	CG (n = 70)	Global test (p_{FDR})	Post-hoc test (p)
**Whole-brain functional connectome**					
CC (×10³)	8.802 ± 0.415	8.753 ± 0.322	8.946 ± 0.437	0.237	–
E_{glob} (×10³)	1.258 ± 0.044	1.255 ± 0.040	1.257 ± 0.059	0.709	–
E_{loc} (×10³)	1.332 ± 0.068	1.326 ± 0.056	1.339 ± 0.081	0.819	–
L	7.976 ± 0.597	8.035 ± 0.726	7.912 ± 0.676	0.819	–
Q (×10³)	3.256 ± 0.133	3.252 ± 0.104	3.337 ± 0.173	**0.041**	**CG vs ncLBP (.019)**<br>**CG vs cLBP (.009)**<br>ncLBP vs cLBP (.588)
**Functional default mode network (fDMN)**					
CC (×10³)	9.186 ± 0.874	9.163 ± 0.720	9.524 ± 0.844	0.228	–
E_{glob} (×10³)	1.057 ± 0.077	1.068 ± 0.071	1.053 ± 0.078	0.258	–
E_{loc} (×10³)	1.103 ± 0.086	1.115 ± 0.084	1.114 ± 0.092	0.678	–
L	9.935 ± 0.74	9.833 ± 0.671	10.023 ± 0.805	0.137	–
Q (×10³)	8.761 ± 0.739	8.851 ± 0.639	9.088 ± 0.736	0.080	–
**Functional somatomotor network (fSMN)**					

CC ($\times 10^3$)	9.544 ± 1.156	9.680 ± 1.124	10.17 ± 1.411	0.118	–
E_{glob} ($\times 10^3$)	1.161 ± 0.093	1.153 ± 0.084	1.161 ± 0.09	0.590	–
E_{loc} ($\times 10^3$)	1.224 ± 0.108	1.222 ± 0.104	1.25 ± 0.108	0.802	–
L	9.158 ± 0.769	9.236 ± 0.730	9.197 ± 0.755	0.590	–
Q ($\times 10^3$)	9.451 ± 0.953	9.653 ± 0.873	9.858 ± 1.157	0.368	–
**Functional vental attention network (fVAN)**					
CC ($\times 10^3$)	8.365 ± 1.361	8.549 ± 1.049	8.829 ± 1.305	0.085	–
E_{glob} ($\times 10^3$)	1.107 ± 0.093	1.099 ± 0.065	1.088 ± 0.09	0.085	CG vs ncLBP (.094)<br>**CG vs cLBP (.019)**<br>ncLBP vs cLBP (.549)
E_{loc} ($\times 10^3$)	1.036 ± 0.094	1.035 ± 0.079	1.032 ± 0.097	0.443	–
L	10.04 ± 0.838	10.058 ± 0.588	10.268 ± 0.937	0.081	**CG vs ncLBP (.027)**<br>**CG vs cLBP (.013)**<br>ncLBP vs cLBP (.851)
Q ($\times 10^3$)	9.35 ± 0.656	9.383 ± 0.761	9.479 ± 0.748	0.639	–

Note. Mean ± SD, not adjusted for age, sex, and scanner. Bold text indicates significant results. CC, clustering coefficient; CG, pain-free control group; E_{glob}, global efficiency; E_{loc}, local efficiency; L, characteristic path length; LBP+, low back pain with pain during scanning; LBP–, low back pain without pain during scanning; Q, modularity.

6 Results – Structure-function coupling (SFC)

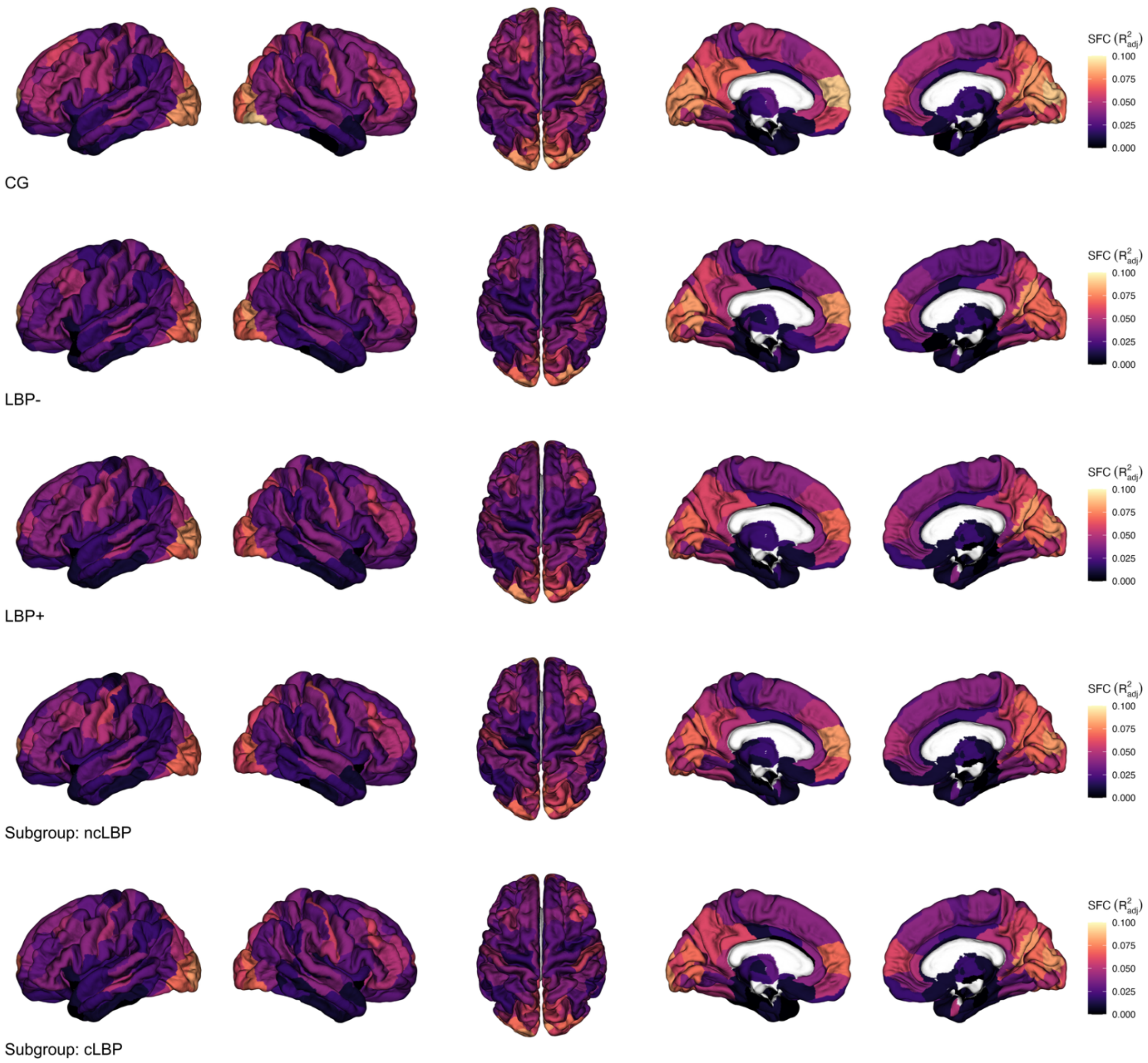

Figure S5: *Structure-function coupling (SFC) per group and subgroup. Higher R^2_{adj} represents stronger coupling. Subcortical regions were projected from volumetric to surface space and may be anatomically distorted. In line with established brain organisation, SFC was strongest in unimodal cortical regions and lower in transmodal association regions.*

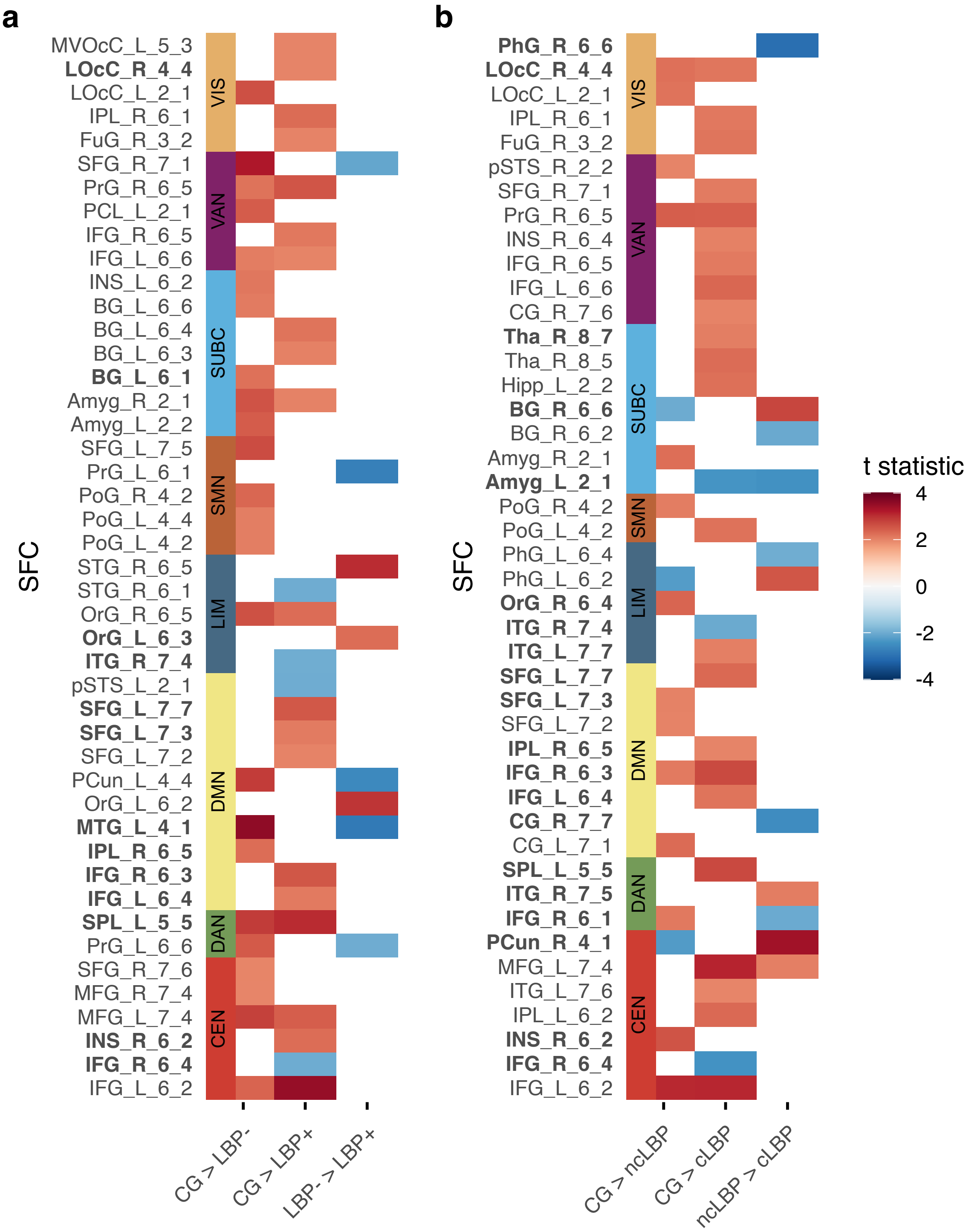

Figure S6: *This figure provides an alternative visualisation of the results presented in Figure 4b, d: Exploratory analysis of region-wise differences in structure-function coupling (SFC) across (**a**) the primary groups (CG, LBP–, LBP+) and (**b**) the subgroups (CG, ncLBP, cLBP). Coloured tiles indicate statistically significant between-group differences. No correction for multiple comparisons was applied. Bold y-axis text indicates a significant correlation to any clinical variable (see Fig. 4e).*

Table S9: *Group comparisons of structure-function coupling for the main and the subgroups across the global and network-wise level.*

**Main groups**					
**SFC**	**CG**	**LBP–**	**LBP+**	**Global test (p_{FDR})**	**Post-hoc test (p)**
Global	0.039 ± 0.010	0.034 ± 0.010	0.035 ± 0.009	**<0.001**	CG vs LBP– **(0.027)**<br>CG vs LBP+ (0.056)<br>LBP– vs LBP+ (0.430)
DMN	0.050 ± 0.012	0.044 ± 0.013	0.044 ± 0.012	**0.002**	**CG vs LBP– (0.031)**<br>**CG vs LBP+ (0.046)**<br>LBP– vs LBP+ (0.540)
VAN	0.034 ± 0.012	0.027 ± 0.011	0.028 ± 0.010	**<0.001**	**CG vs LBP– (0.005)**<br>**CG vs LBP+ (0.014)**<br>LBP– vs LBP+ (0.353)
SMN	0.043 ± 0.015	0.037 ± 0.016	0.038 ± 0.014	**0.027**	CG vs LBP– (0.097)<br>CG vs LBP+ (0.569)<br>LBP– vs LBP+ (0.141)
**Subgroups**					
**SFC**	**CG**	**ncLBP**	**cLBP**	**Global test (p_{FDR})**	**Post-hoc test (p)**
Global	0.039 ± 0.010	0.034 ± 0.008	0.034 ± 0.009	**<0.001**	CG vs ncLBP (0.097)<br>**CG vs cLBP (0.044)**<br>ncLBP vs cLBP (0.958)
DMN	0.050 ± 0.012	0.043 ± 0.009	0.044 ± 0.012	**0.004**	**CG vs ncLBP (0.041)**<br>CG vs cLBP (0.053)<br>ncLBP vs cLBP (0.576)
VAN	0.034 ± 0.012	0.028 ± 0.009	0.027 ± 0.010	**<0.001**	**CG vs ncLBP (0.047)**<br>**CG vs cLBP (0.006)**<br>ncLBP vs cLBP (0.816)
SMN	0.043 ± 0.015	0.039 ± 0.014	0.038 ± 0.014	0.059	-
Note. Mean ± SD, not adjusted for age, sex, and scanner. CG, pain-free control group; cLBP, chronic low back pain; ncLBP, non-chronic low back pain; DMN, default mode network; VAN, ventral attention network; SMN, somatomotor network; SFC, structure-function coupling.					

Table S10: *Unadjusted regional structure-function coupling (SFC) values for all groups.*

SFC	CG	LBP-	LBP+	ncLBP	cLBP
SFG_L_7_1	0.047 ± 0.033	0.040 ± 0.031	0.045 ± 0.033	0.041 ± 0.032	0.044 ± 0.035
SFG_R_7_1	0.055 ± 0.035	0.034 ± 0.030	0.043 ± 0.033	0.044 ± 0.031	0.041 ± 0.032
SFG_L_7_2	0.062 ± 0.038	0.049 ± 0.034	0.049 ± 0.034	0.047 ± 0.031	0.050 ± 0.035
SFG_R_7_2	0.049 ± 0.036	0.040 ± 0.027	0.041 ± 0.031	0.038 ± 0.030	0.043 ± 0.031
SFG_L_7_3	0.052 ± 0.039	0.043 ± 0.033	0.040 ± 0.031	0.039 ± 0.029	0.041 ± 0.032
SFG_R_7_3	0.042 ± 0.030	0.036 ± 0.025	0.035 ± 0.028	0.032 ± 0.031	0.038 ± 0.028
SFG_L_7_4	0.039 ± 0.035	0.030 ± 0.027	0.032 ± 0.028	0.027 ± 0.028	0.033 ± 0.027
SFG_R_7_4	0.041 ± 0.030	0.037 ± 0.031	0.032 ± 0.027	0.035 ± 0.030	0.031 ± 0.024
SFG_L_7_5	0.054 ± 0.037	0.037 ± 0.037	0.044 ± 0.032	0.042 ± 0.032	0.042 ± 0.031
SFG_R_7_5	0.045 ± 0.036	0.036 ± 0.027	0.040 ± 0.032	0.040 ± 0.033	0.039 ± 0.033

SFG_L_7_6	0.055 ± 0.031	0.044 ± 0.032	0.051 ± 0.035	0.057 ± 0.038	0.049 ± 0.037
SFG_R_7_6	0.055 ± 0.035	0.041 ± 0.030	0.047 ± 0.032	0.048 ± 0.035	0.048 ± 0.032
SFG_L_7_7	0.095 ± 0.043	0.081 ± 0.037	0.078 ± 0.043	0.081 ± 0.041	0.078 ± 0.043
SFG_R_7_7	0.064 ± 0.032	0.063 ± 0.038	0.058 ± 0.036	0.055 ± 0.037	0.061 ± 0.036
MFG_L_7_1	0.041 ± 0.027	0.038 ± 0.027	0.037 ± 0.030	0.040 ± 0.029	0.034 ± 0.028
MFG_R_7_1	0.050 ± 0.037	0.048 ± 0.033	0.049 ± 0.035	0.054 ± 0.036	0.048 ± 0.033
MFG_L_7_2	0.059 ± 0.042	0.050 ± 0.035	0.049 ± 0.032	0.051 ± 0.035	0.048 ± 0.031
MFG_R_7_2	0.044 ± 0.033	0.038 ± 0.028	0.045 ± 0.032	0.050 ± 0.036	0.043 ± 0.030
MFG_L_7_3	0.053 ± 0.034	0.044 ± 0.029	0.050 ± 0.031	0.053 ± 0.030	0.050 ± 0.032
MFG_R_7_3	0.061 ± 0.034	0.057 ± 0.034	0.056 ± 0.037	0.055 ± 0.043	0.056 ± 0.036
MFG_L_7_4	0.052 ± 0.032	0.037 ± 0.028	0.041 ± 0.026	0.049 ± 0.030	0.038 ± 0.025
MFG_R_7_4	0.068 ± 0.036	0.054 ± 0.034	0.058 ± 0.035	0.055 ± 0.036	0.059 ± 0.035
MFG_L_7_5	0.057 ± 0.034	0.052 ± 0.033	0.053 ± 0.035	0.056 ± 0.031	0.053 ± 0.038
MFG_R_7_5	0.067 ± 0.033	0.058 ± 0.037	0.063 ± 0.034	0.065 ± 0.032	0.063 ± 0.037
MFG_L_7_6	0.041 ± 0.030	0.032 ± 0.028	0.037 ± 0.029	0.032 ± 0.028	0.038 ± 0.029
MFG_R_7_6	0.040 ± 0.029	0.039 ± 0.025	0.036 ± 0.027	0.040 ± 0.026	0.034 ± 0.028
MFG_L_7_7	0.034 ± 0.024	0.030 ± 0.025	0.031 ± 0.029	0.036 ± 0.034	0.030 ± 0.028
MFG_R_7_7	0.044 ± 0.035	0.046 ± 0.034	0.040 ± 0.031	0.038 ± 0.034	0.040 ± 0.030
IFG_L_6_1	0.041 ± 0.027	0.032 ± 0.027	0.037 ± 0.029	0.037 ± 0.029	0.034 ± 0.026
IFG_R_6_1	0.044 ± 0.034	0.035 ± 0.031	0.036 ± 0.028	0.030 ± 0.026	0.040 ± 0.029
IFG_L_6_2	0.058 ± 0.039	0.044 ± 0.028	0.041 ± 0.029	0.039 ± 0.031	0.042 ± 0.028
IFG_R_6_2	0.034 ± 0.028	0.033 ± 0.026	0.033 ± 0.028	0.031 ± 0.023	0.034 ± 0.029
IFG_L_6_3	0.047 ± 0.035	0.044 ± 0.031	0.043 ± 0.032	0.035 ± 0.025	0.046 ± 0.034
IFG_R_6_3	0.056 ± 0.036	0.045 ± 0.031	0.040 ± 0.036	0.041 ± 0.031	0.039 ± 0.032
IFG_L_6_4	0.051 ± 0.032	0.043 ± 0.034	0.041 ± 0.031	0.042 ± 0.034	0.040 ± 0.031
IFG_R_6_4	0.032 ± 0.026	0.038 ± 0.028	0.039 ± 0.030	0.035 ± 0.031	0.041 ± 0.029
IFG_L_6_5	0.044 ± 0.032	0.036 ± 0.032	0.034 ± 0.029	0.030 ± 0.028	0.033 ± 0.029
IFG_R_6_5	0.053 ± 0.035	0.044 ± 0.030	0.041 ± 0.032	0.039 ± 0.027	0.040 ± 0.034
IFG_L_6_6	0.047 ± 0.031	0.035 ± 0.028	0.036 ± 0.029	0.035 ± 0.028	0.034 ± 0.029
IFG_R_6_6	0.035 ± 0.027	0.035 ± 0.027	0.037 ± 0.030	0.032 ± 0.028	0.039 ± 0.030
OrG_L_6_1	0.061 ± 0.039	0.055 ± 0.035	0.057 ± 0.036	0.060 ± 0.033	0.057 ± 0.039
OrG_R_6_1	0.054 ± 0.030	0.050 ± 0.036	0.045 ± 0.034	0.047 ± 0.030	0.046 ± 0.037
OrG_L_6_2	0.034 ± 0.025	0.038 ± 0.029	0.027 ± 0.021	0.031 ± 0.020	0.027 ± 0.022
OrG_R_6_2	0.040 ± 0.028	0.039 ± 0.029	0.037 ± 0.029	0.039 ± 0.032	0.038 ± 0.028
OrG_L_6_3	0.034 ± 0.026	0.035 ± 0.032	0.026 ± 0.025	0.022 ± 0.024	0.027 ± 0.026
OrG_R_6_3	0.043 ± 0.029	0.043 ± 0.031	0.037 ± 0.033	0.035 ± 0.028	0.039 ± 0.032
OrG_L_6_4	0.023 ± 0.024	0.028 ± 0.032	0.024 ± 0.027	0.027 ± 0.026	0.023 ± 0.027
OrG_R_6_4	0.027 ± 0.026	0.027 ± 0.026	0.020 ± 0.027	0.014 ± 0.019	0.022 ± 0.029
OrG_L_6_5	0.018 ± 0.023	0.020 ± 0.028	0.018 ± 0.024	0.015 ± 0.021	0.018 ± 0.023
OrG_R_6_5	0.018 ± 0.021	0.008 ± 0.021	0.010 ± 0.017	0.010 ± 0.018	0.011 ± 0.018
OrG_L_6_6	0.050 ± 0.030	0.043 ± 0.027	0.044 ± 0.031	0.043 ± 0.026	0.042 ± 0.032
OrG_R_6_6	0.054 ± 0.028	0.047 ± 0.029	0.049 ± 0.034	0.047 ± 0.030	0.052 ± 0.037
PrG_L_6_1	0.050 ± 0.031	0.040 ± 0.030	0.051 ± 0.030	0.056 ± 0.030	0.046 ± 0.028
PrG_R_6_1	0.041 ± 0.030	0.042 ± 0.029	0.045 ± 0.033	0.042 ± 0.029	0.046 ± 0.034

PrG_L_6_2	0.040 ± 0.039	0.028 ± 0.022	0.032 ± 0.032	0.032 ± 0.025	0.033 ± 0.036
PrG_R_6_2	0.033 ± 0.027	0.030 ± 0.025	0.026 ± 0.025	0.027 ± 0.025	0.026 ± 0.026
PrG_L_6_3	0.035 ± 0.031	0.029 ± 0.032	0.028 ± 0.028	0.024 ± 0.029	0.029 ± 0.027
PrG_R_6_3	0.044 ± 0.029	0.036 ± 0.032	0.036 ± 0.030	0.036 ± 0.031	0.036 ± 0.031
PrG_L_6_4	0.032 ± 0.043	0.024 ± 0.026	0.022 ± 0.023	0.018 ± 0.022	0.024 ± 0.025
PrG_R_6_4	0.044 ± 0.038	0.032 ± 0.032	0.038 ± 0.034	0.041 ± 0.028	0.039 ± 0.037
PrG_L_6_5	0.036 ± 0.028	0.028 ± 0.025	0.031 ± 0.026	0.035 ± 0.027	0.029 ± 0.025
PrG_R_6_5	0.049 ± 0.030	0.036 ± 0.034	0.035 ± 0.029	0.033 ± 0.028	0.035 ± 0.028
PrG_L_6_6	0.052 ± 0.033	0.037 ± 0.026	0.044 ± 0.032	0.046 ± 0.028	0.044 ± 0.032
PrG_R_6_6	0.040 ± 0.032	0.038 ± 0.027	0.040 ± 0.031	0.037 ± 0.032	0.038 ± 0.030
PCL_L_2_1	0.040 ± 0.030	0.024 ± 0.030	0.032 ± 0.031	0.027 ± 0.026	0.034 ± 0.032
PCL_R_2_1	0.042 ± 0.036	0.032 ± 0.030	0.036 ± 0.030	0.033 ± 0.032	0.036 ± 0.030
PCL_L_2_2	0.054 ± 0.044	0.040 ± 0.041	0.040 ± 0.035	0.038 ± 0.037	0.040 ± 0.033
PCL_R_2_2	0.049 ± 0.039	0.036 ± 0.035	0.042 ± 0.039	0.043 ± 0.039	0.040 ± 0.039
STG_L_6_1	0.015 ± 0.021	0.016 ± 0.032	0.010 ± 0.019	0.011 ± 0.018	0.010 ± 0.017
STG_R_6_1	0.008 ± 0.017	0.013 ± 0.019	0.012 ± 0.020	0.013 ± 0.020	0.011 ± 0.019
STG_L_6_2	0.036 ± 0.029	0.037 ± 0.030	0.036 ± 0.032	0.035 ± 0.032	0.038 ± 0.033
STG_R_6_2	0.033 ± 0.031	0.033 ± 0.029	0.035 ± 0.031	0.032 ± 0.030	0.035 ± 0.032
STG_L_6_3	0.039 ± 0.036	0.031 ± 0.025	0.030 ± 0.027	0.028 ± 0.029	0.030 ± 0.025
STG_R_6_3	0.036 ± 0.027	0.038 ± 0.030	0.035 ± 0.029	0.038 ± 0.033	0.031 ± 0.029
STG_L_6_4	0.049 ± 0.043	0.043 ± 0.035	0.050 ± 0.040	0.047 ± 0.038	0.049 ± 0.039
STG_R_6_4	0.046 ± 0.038	0.038 ± 0.033	0.044 ± 0.036	0.045 ± 0.033	0.043 ± 0.037
STG_L_6_5	0.015 ± 0.027	0.010 ± 0.016	0.011 ± 0.020	0.014 ± 0.022	0.011 ± 0.021
STG_R_6_5	0.015 ± 0.022	0.020 ± 0.024	0.011 ± 0.019	0.010 ± 0.016	0.011 ± 0.020
STG_L_6_6	0.026 ± 0.030	0.022 ± 0.026	0.025 ± 0.027	0.030 ± 0.030	0.023 ± 0.025
STG_R_6_6	0.034 ± 0.036	0.026 ± 0.030	0.030 ± 0.027	0.030 ± 0.026	0.030 ± 0.028
MTG_L_4_1	0.043 ± 0.032	0.023 ± 0.024	0.034 ± 0.029	0.031 ± 0.027	0.035 ± 0.030
MTG_R_4_1	0.021 ± 0.023	0.021 ± 0.021	0.019 ± 0.025	0.022 ± 0.025	0.017 ± 0.023
MTG_L_4_2	0.035 ± 0.030	0.032 ± 0.032	0.029 ± 0.030	0.031 ± 0.033	0.028 ± 0.030
MTG_R_4_2	0.029 ± 0.035	0.029 ± 0.028	0.026 ± 0.028	0.026 ± 0.028	0.025 ± 0.028
MTG_L_4_3	0.038 ± 0.029	0.031 ± 0.026	0.033 ± 0.030	0.031 ± 0.035	0.034 ± 0.029
MTG_R_4_3	0.033 ± 0.031	0.027 ± 0.025	0.027 ± 0.027	0.028 ± 0.026	0.026 ± 0.029
MTG_L_4_4	0.054 ± 0.039	0.054 ± 0.038	0.054 ± 0.036	0.054 ± 0.034	0.054 ± 0.036
MTG_R_4_4	0.047 ± 0.033	0.044 ± 0.035	0.043 ± 0.032	0.044 ± 0.030	0.042 ± 0.032
ITG_L_7_1	0.013 ± 0.022	0.011 ± 0.017	0.014 ± 0.021	0.008 ± 0.018	0.015 ± 0.021
ITG_R_7_1	0.010 ± 0.024	0.013 ± 0.024	0.017 ± 0.026	0.017 ± 0.029	0.017 ± 0.025
ITG_L_7_2	0.033 ± 0.031	0.037 ± 0.029	0.033 ± 0.026	0.031 ± 0.023	0.033 ± 0.026
ITG_R_7_2	0.020 ± 0.022	0.018 ± 0.024	0.017 ± 0.022	0.017 ± 0.019	0.017 ± 0.022
ITG_L_7_3	0.020 ± 0.038	0.023 ± 0.049	0.017 ± 0.038	0.028 ± 0.055	0.014 ± 0.034
ITG_R_7_3	0.013 ± 0.017	0.020 ± 0.026	0.019 ± 0.034	0.022 ± 0.039	0.018 ± 0.032
ITG_L_7_4	0.022 ± 0.040	0.012 ± 0.022	0.015 ± 0.032	0.016 ± 0.038	0.013 ± 0.025
ITG_R_7_4	0.006 ± 0.017	0.011 ± 0.017	0.012 ± 0.024	0.011 ± 0.029	0.013 ± 0.021
ITG_L_7_5	0.035 ± 0.030	0.035 ± 0.032	0.030 ± 0.026	0.037 ± 0.030	0.029 ± 0.026
ITG_R_7_5	0.026 ± 0.026	0.025 ± 0.024	0.021 ± 0.024	0.028 ± 0.019	0.019 ± 0.023

ITG_L_7_6	0.021 ± 0.022	0.020 ± 0.028	0.016 ± 0.022	0.017 ± 0.027	0.014 ± 0.020
ITG_R_7_6	0.030 ± 0.026	0.020 ± 0.025	0.024 ± 0.026	0.026 ± 0.025	0.024 ± 0.026
ITG_L_7_7	0.016 ± 0.024	0.013 ± 0.023	0.011 ± 0.022	0.013 ± 0.027	0.009 ± 0.019
ITG_R_7_7	0.010 ± 0.018	0.010 ± 0.019	0.010 ± 0.018	0.008 ± 0.014	0.012 ± 0.020
FuG_L_3_1	0.020 ± 0.026	0.016 ± 0.021	0.017 ± 0.026	0.014 ± 0.023	0.017 ± 0.025
FuG_R_3_1	0.015 ± 0.024	0.019 ± 0.024	0.017 ± 0.026	0.017 ± 0.021	0.016 ± 0.028
FuG_L_3_2	0.055 ± 0.036	0.055 ± 0.041	0.052 ± 0.036	0.055 ± 0.040	0.050 ± 0.036
FuG_R_3_2	0.062 ± 0.045	0.053 ± 0.041	0.047 ± 0.032	0.046 ± 0.035	0.045 ± 0.030
FuG_L_3_3	0.039 ± 0.037	0.042 ± 0.031	0.036 ± 0.031	0.038 ± 0.033	0.034 ± 0.031
FuG_R_3_3	0.033 ± 0.028	0.034 ± 0.025	0.029 ± 0.029	0.027 ± 0.029	0.029 ± 0.028
PhG_L_6_1	0.013 ± 0.027	0.013 ± 0.038	0.013 ± 0.032	0.017 ± 0.038	0.014 ± 0.034
PhG_R_6_1	0.011 ± 0.024	0.016 ± 0.039	0.011 ± 0.031	0.013 ± 0.034	0.011 ± 0.031
PhG_L_6_2	0.009 ± 0.017	0.009 ± 0.016	0.010 ± 0.019	0.017 ± 0.024	0.009 ± 0.017
PhG_R_6_2	0.007 ± 0.016	0.010 ± 0.017	0.007 ± 0.018	0.008 ± 0.024	0.005 ± 0.015
PhG_L_6_3	0.011 ± 0.020	0.012 ± 0.020	0.015 ± 0.020	0.016 ± 0.020	0.013 ± 0.020
PhG_R_6_3	0.012 ± 0.021	0.009 ± 0.017	0.011 ± 0.017	0.011 ± 0.015	0.012 ± 0.018
PhG_L_6_4	0.010 ± 0.016	0.008 ± 0.016	0.008 ± 0.017	0.004 ± 0.014	0.009 ± 0.018
PhG_R_6_4	0.009 ± 0.020	0.004 ± 0.014	0.008 ± 0.019	0.006 ± 0.016	0.006 ± 0.018
PhG_L_6_5	0.012 ± 0.031	0.030 ± 0.055	0.021 ± 0.046	0.033 ± 0.053	0.019 ± 0.044
PhG_R_6_5	0.028 ± 0.052	0.038 ± 0.054	0.048 ± 0.064	0.043 ± 0.064	0.052 ± 0.066
PhG_L_6_6	0.017 ± 0.022	0.013 ± 0.022	0.012 ± 0.021	0.013 ± 0.020	0.012 ± 0.022
PhG_R_6_6	0.010 ± 0.019	0.010 ± 0.021	0.009 ± 0.017	0.002 ± 0.013	0.012 ± 0.019
pSTS_L_2_1	0.033 ± 0.027	0.035 ± 0.030	0.038 ± 0.032	0.035 ± 0.020	0.037 ± 0.034
pSTS_R_2_1	0.032 ± 0.027	0.034 ± 0.035	0.032 ± 0.028	0.029 ± 0.027	0.031 ± 0.028
pSTS_L_2_2	0.031 ± 0.026	0.028 ± 0.028	0.027 ± 0.026	0.026 ± 0.026	0.027 ± 0.025
pSTS_R_2_2	0.031 ± 0.028	0.031 ± 0.027	0.025 ± 0.024	0.021 ± 0.021	0.024 ± 0.022
SPL_L_5_1	0.050 ± 0.033	0.047 ± 0.033	0.048 ± 0.035	0.047 ± 0.034	0.048 ± 0.033
SPL_R_5_1	0.052 ± 0.033	0.042 ± 0.026	0.049 ± 0.034	0.052 ± 0.031	0.047 ± 0.036
SPL_L_5_2	0.071 ± 0.036	0.062 ± 0.036	0.063 ± 0.038	0.062 ± 0.040	0.062 ± 0.038
SPL_R_5_2	0.064 ± 0.039	0.062 ± 0.037	0.065 ± 0.037	0.064 ± 0.034	0.063 ± 0.039
SPL_L_5_3	0.044 ± 0.029	0.045 ± 0.039	0.041 ± 0.030	0.041 ± 0.034	0.038 ± 0.028
SPL_R_5_3	0.044 ± 0.032	0.042 ± 0.032	0.042 ± 0.033	0.046 ± 0.029	0.040 ± 0.035
SPL_L_5_4	0.039 ± 0.027	0.040 ± 0.030	0.041 ± 0.032	0.045 ± 0.039	0.040 ± 0.031
SPL_R_5_4	0.050 ± 0.035	0.043 ± 0.034	0.041 ± 0.035	0.042 ± 0.031	0.040 ± 0.036
SPL_L_5_5	0.043 ± 0.029	0.028 ± 0.026	0.029 ± 0.028	0.030 ± 0.025	0.028 ± 0.029
SPL_R_5_5	0.037 ± 0.034	0.029 ± 0.024	0.035 ± 0.027	0.037 ± 0.025	0.035 ± 0.027
IPL_L_6_1	0.052 ± 0.029	0.054 ± 0.037	0.051 ± 0.033	0.047 ± 0.033	0.051 ± 0.033
IPL_R_6_1	0.049 ± 0.032	0.038 ± 0.027	0.037 ± 0.027	0.039 ± 0.028	0.037 ± 0.028
IPL_L_6_2	0.032 ± 0.024	0.027 ± 0.026	0.027 ± 0.023	0.031 ± 0.025	0.024 ± 0.021
IPL_R_6_2	0.037 ± 0.029	0.037 ± 0.027	0.034 ± 0.024	0.041 ± 0.023	0.034 ± 0.025
IPL_L_6_3	0.041 ± 0.024	0.041 ± 0.031	0.042 ± 0.031	0.041 ± 0.033	0.040 ± 0.029
IPL_R_6_3	0.055 ± 0.029	0.050 ± 0.031	0.053 ± 0.032	0.054 ± 0.032	0.050 ± 0.032
IPL_L_6_4	0.025 ± 0.026	0.028 ± 0.024	0.026 ± 0.024	0.029 ± 0.023	0.025 ± 0.026
IPL_R_6_4	0.031 ± 0.021	0.031 ± 0.022	0.026 ± 0.022	0.030 ± 0.027	0.024 ± 0.021

IPL_L_6_5	0.039 ± 0.029	0.033 ± 0.028	0.032 ± 0.024	0.028 ± 0.023	0.033 ± 0.024
IPL_R_6_5	0.051 ± 0.032	0.039 ± 0.023	0.043 ± 0.028	0.042 ± 0.029	0.042 ± 0.026
IPL_L_6_6	0.040 ± 0.028	0.039 ± 0.029	0.040 ± 0.029	0.042 ± 0.027	0.037 ± 0.029
IPL_R_6_6	0.034 ± 0.023	0.038 ± 0.029	0.039 ± 0.029	0.038 ± 0.029	0.040 ± 0.030
PCun_L_4_1	0.066 ± 0.032	0.065 ± 0.036	0.061 ± 0.034	0.059 ± 0.032	0.062 ± 0.035
PCun_R_4_1	0.071 ± 0.031	0.070 ± 0.036	0.067 ± 0.038	0.082 ± 0.039	0.061 ± 0.034
PCun_L_4_2	0.042 ± 0.028	0.044 ± 0.033	0.041 ± 0.032	0.043 ± 0.032	0.041 ± 0.033
PCun_R_4_2	0.055 ± 0.034	0.050 ± 0.037	0.056 ± 0.034	0.053 ± 0.032	0.060 ± 0.033
PCun_L_4_3	0.075 ± 0.033	0.067 ± 0.036	0.070 ± 0.037	0.071 ± 0.044	0.069 ± 0.036
PCun_R_4_3	0.084 ± 0.042	0.080 ± 0.039	0.083 ± 0.040	0.080 ± 0.049	0.081 ± 0.036
PCun_L_4_4	0.071 ± 0.034	0.054 ± 0.030	0.066 ± 0.034	0.065 ± 0.034	0.068 ± 0.036
PCun_R_4_4	0.064 ± 0.035	0.059 ± 0.032	0.065 ± 0.037	0.062 ± 0.039	0.065 ± 0.037
PoG_L_4_1	0.054 ± 0.034	0.047 ± 0.034	0.056 ± 0.036	0.062 ± 0.036	0.053 ± 0.035
PoG_R_4_1	0.071 ± 0.039	0.061 ± 0.041	0.068 ± 0.036	0.075 ± 0.034	0.061 ± 0.036
PoG_L_4_2	0.054 ± 0.035	0.040 ± 0.032	0.042 ± 0.032	0.050 ± 0.039	0.040 ± 0.029
PoG_R_4_2	0.060 ± 0.035	0.044 ± 0.032	0.049 ± 0.031	0.043 ± 0.031	0.050 ± 0.031
PoG_L_4_3	0.037 ± 0.030	0.031 ± 0.026	0.030 ± 0.025	0.028 ± 0.026	0.030 ± 0.025
PoG_R_4_3	0.044 ± 0.033	0.039 ± 0.029	0.039 ± 0.028	0.042 ± 0.030	0.036 ± 0.028
PoG_L_4_4	0.057 ± 0.043	0.041 ± 0.035	0.043 ± 0.036	0.044 ± 0.037	0.043 ± 0.035
PoG_R_4_4	0.039 ± 0.033	0.039 ± 0.036	0.037 ± 0.033	0.035 ± 0.032	0.039 ± 0.034
INS_L_6_1	0.021 ± 0.021	0.024 ± 0.025	0.023 ± 0.023	0.022 ± 0.017	0.021 ± 0.024
INS_R_6_1	0.016 ± 0.021	0.014 ± 0.021	0.014 ± 0.020	0.017 ± 0.022	0.013 ± 0.019
INS_L_6_2	0.013 ± 0.021	0.005 ± 0.013	0.009 ± 0.018	0.008 ± 0.017	0.009 ± 0.018
INS_R_6_2	0.015 ± 0.022	0.011 ± 0.017	0.008 ± 0.018	0.005 ± 0.018	0.009 ± 0.019
INS_L_6_3	0.024 ± 0.024	0.018 ± 0.022	0.021 ± 0.024	0.022 ± 0.027	0.020 ± 0.022
INS_R_6_3	0.031 ± 0.028	0.028 ± 0.028	0.025 ± 0.025	0.024 ± 0.026	0.026 ± 0.024
INS_L_6_4	0.011 ± 0.023	0.011 ± 0.019	0.012 ± 0.019	0.018 ± 0.023	0.011 ± 0.018
INS_R_6_4	0.016 ± 0.026	0.013 ± 0.022	0.010 ± 0.018	0.013 ± 0.020	0.009 ± 0.017
INS_L_6_5	0.032 ± 0.027	0.025 ± 0.028	0.024 ± 0.027	0.027 ± 0.023	0.020 ± 0.025
INS_R_6_5	0.023 ± 0.021	0.025 ± 0.025	0.023 ± 0.025	0.026 ± 0.025	0.020 ± 0.024
INS_L_6_6	0.030 ± 0.028	0.021 ± 0.032	0.023 ± 0.025	0.026 ± 0.018	0.022 ± 0.026
INS_R_6_6	0.036 ± 0.032	0.028 ± 0.024	0.028 ± 0.029	0.025 ± 0.024	0.029 ± 0.029
CG_L_7_1	0.071 ± 0.039	0.056 ± 0.040	0.062 ± 0.035	0.052 ± 0.028	0.063 ± 0.036
CG_R_7_1	0.059 ± 0.032	0.058 ± 0.039	0.055 ± 0.032	0.050 ± 0.029	0.053 ± 0.032
CG_L_7_2	0.012 ± 0.021	0.008 ± 0.014	0.010 ± 0.017	0.009 ± 0.018	0.009 ± 0.015
CG_R_7_2	0.016 ± 0.020	0.013 ± 0.017	0.013 ± 0.020	0.012 ± 0.018	0.012 ± 0.020
CG_L_7_3	0.050 ± 0.033	0.044 ± 0.032	0.049 ± 0.036	0.047 ± 0.034	0.047 ± 0.035
CG_R_7_3	0.036 ± 0.028	0.032 ± 0.033	0.035 ± 0.029	0.032 ± 0.025	0.037 ± 0.031
CG_L_7_4	0.067 ± 0.042	0.054 ± 0.035	0.051 ± 0.040	0.050 ± 0.041	0.051 ± 0.041
CG_R_7_4	0.060 ± 0.035	0.052 ± 0.036	0.047 ± 0.033	0.048 ± 0.032	0.046 ± 0.035
CG_L_7_5	0.024 ± 0.028	0.021 ± 0.022	0.026 ± 0.025	0.028 ± 0.024	0.022 ± 0.022
CG_R_7_5	0.021 ± 0.027	0.017 ± 0.022	0.020 ± 0.027	0.021 ± 0.029	0.020 ± 0.026
CG_L_7_6	0.024 ± 0.022	0.020 ± 0.026	0.020 ± 0.025	0.023 ± 0.023	0.019 ± 0.026
CG_R_7_6	0.030 ± 0.026	0.027 ± 0.026	0.022 ± 0.023	0.026 ± 0.024	0.021 ± 0.023

CG_L_7_7	0.051 ± 0.038	0.049 ± 0.037	0.046 ± 0.031	0.047 ± 0.027	0.047 ± 0.033
CG_R_7_7	0.049 ± 0.027	0.048 ± 0.038	0.048 ± 0.034	0.040 ± 0.028	0.053 ± 0.033
MVOcC_L_5_1	0.058 ± 0.044	0.054 ± 0.036	0.046 ± 0.037	0.049 ± 0.040	0.046 ± 0.037
MVOcC_R_5_1	0.064 ± 0.045	0.054 ± 0.039	0.053 ± 0.038	0.052 ± 0.043	0.054 ± 0.037
MVOcC_L_5_2	0.085 ± 0.049	0.089 ± 0.055	0.079 ± 0.051	0.076 ± 0.050	0.077 ± 0.048
MVOcC_R_5_2	0.090 ± 0.053	0.077 ± 0.050	0.078 ± 0.050	0.070 ± 0.052	0.075 ± 0.049
MVOcC_L_5_3	0.080 ± 0.049	0.077 ± 0.051	0.063 ± 0.043	0.057 ± 0.040	0.062 ± 0.045
MVOcC_R_5_3	0.094 ± 0.051	0.080 ± 0.045	0.084 ± 0.050	0.088 ± 0.054	0.080 ± 0.050
MVOcC_L_5_4	0.043 ± 0.036	0.037 ± 0.030	0.038 ± 0.030	0.037 ± 0.030	0.037 ± 0.029
MVOcC_R_5_4	0.043 ± 0.039	0.034 ± 0.031	0.038 ± 0.034	0.039 ± 0.037	0.037 ± 0.034
MVOcC_L_5_5	0.070 ± 0.046	0.062 ± 0.042	0.062 ± 0.039	0.063 ± 0.039	0.058 ± 0.037
MVOcC_R_5_5	0.071 ± 0.044	0.065 ± 0.045	0.065 ± 0.042	0.059 ± 0.042	0.064 ± 0.043
LOcC_L_4_1	0.081 ± 0.042	0.077 ± 0.048	0.081 ± 0.042	0.078 ± 0.041	0.078 ± 0.042
LOcC_R_4_1	0.085 ± 0.043	0.083 ± 0.041	0.076 ± 0.038	0.076 ± 0.031	0.074 ± 0.040
LOcC_L_4_2	0.039 ± 0.026	0.031 ± 0.023	0.036 ± 0.030	0.037 ± 0.029	0.034 ± 0.028
LOcC_R_4_2	0.050 ± 0.036	0.048 ± 0.032	0.043 ± 0.035	0.038 ± 0.025	0.044 ± 0.036
LOcC_L_4_3	0.088 ± 0.042	0.083 ± 0.048	0.082 ± 0.044	0.078 ± 0.046	0.081 ± 0.044
LOcC_R_4_3	0.078 ± 0.041	0.071 ± 0.042	0.070 ± 0.039	0.070 ± 0.042	0.066 ± 0.038
LOcC_L_4_4	0.087 ± 0.051	0.078 ± 0.048	0.075 ± 0.047	0.071 ± 0.039	0.074 ± 0.049
LOcC_R_4_4	0.092 ± 0.047	0.079 ± 0.048	0.073 ± 0.042	0.068 ± 0.048	0.071 ± 0.040
LOcC_L_2_1	0.074 ± 0.048	0.053 ± 0.040	0.060 ± 0.042	0.053 ± 0.043	0.059 ± 0.041
LOcC_R_2_1	0.077 ± 0.045	0.060 ± 0.043	0.067 ± 0.043	0.062 ± 0.044	0.063 ± 0.041
LOcC_L_2_2	0.055 ± 0.035	0.045 ± 0.035	0.051 ± 0.035	0.045 ± 0.035	0.052 ± 0.034
LOcC_R_2_2	0.064 ± 0.034	0.056 ± 0.034	0.057 ± 0.034	0.061 ± 0.036	0.055 ± 0.032
Amyg_L_2_1	0.009 ± 0.018	0.009 ± 0.018	0.011 ± 0.018	0.006 ± 0.016	0.014 ± 0.020
Amyg_R_2_1	0.018 ± 0.021	0.009 ± 0.017	0.012 ± 0.019	0.009 ± 0.015	0.012 ± 0.019
Amyg_L_2_2	0.010 ± 0.023	0.002 ± 0.013	0.005 ± 0.016	0.007 ± 0.019	0.006 ± 0.016
Amyg_R_2_2	0.005 ± 0.019	0.006 ± 0.016	0.005 ± 0.015	0.001 ± 0.013	0.005 ± 0.014
Hipp_L_2_1	0.023 ± 0.025	0.015 ± 0.019	0.016 ± 0.022	0.012 ± 0.020	0.017 ± 0.022
Hipp_R_2_1	0.017 ± 0.023	0.015 ± 0.024	0.015 ± 0.019	0.013 ± 0.016	0.016 ± 0.020
Hipp_L_2_2	0.019 ± 0.027	0.017 ± 0.024	0.011 ± 0.018	0.011 ± 0.015	0.010 ± 0.018
Hipp_R_2_2	0.017 ± 0.020	0.010 ± 0.018	0.012 ± 0.020	0.016 ± 0.024	0.012 ± 0.020
BG_L_6_1	0.027 ± 0.026	0.017 ± 0.027	0.021 ± 0.023	0.017 ± 0.022	0.022 ± 0.023
BG_R_6_1	0.021 ± 0.024	0.015 ± 0.019	0.021 ± 0.024	0.027 ± 0.032	0.020 ± 0.022
BG_L_6_2	0.015 ± 0.019	0.011 ± 0.016	0.013 ± 0.020	0.013 ± 0.020	0.012 ± 0.019
BG_R_6_2	0.015 ± 0.024	0.011 ± 0.021	0.011 ± 0.020	0.005 ± 0.017	0.013 ± 0.022
BG_L_6_3	0.019 ± 0.024	0.013 ± 0.021	0.012 ± 0.019	0.013 ± 0.021	0.013 ± 0.020
BG_R_6_3	0.020 ± 0.025	0.016 ± 0.019	0.015 ± 0.021	0.013 ± 0.022	0.015 ± 0.021
BG_L_6_4	0.022 ± 0.030	0.014 ± 0.021	0.015 ± 0.022	0.013 ± 0.021	0.015 ± 0.022
BG_R_6_4	0.020 ± 0.023	0.015 ± 0.021	0.017 ± 0.021	0.014 ± 0.021	0.017 ± 0.022
BG_L_6_5	0.013 ± 0.020	0.011 ± 0.016	0.014 ± 0.020	0.009 ± 0.016	0.015 ± 0.021
BG_R_6_5	0.020 ± 0.023	0.014 ± 0.023	0.016 ± 0.022	0.015 ± 0.023	0.018 ± 0.023
BG_L_6_6	0.027 ± 0.030	0.016 ± 0.020	0.019 ± 0.024	0.015 ± 0.021	0.018 ± 0.023
BG_R_6_6	0.019 ± 0.021	0.015 ± 0.020	0.017 ± 0.023	0.026 ± 0.031	0.015 ± 0.020

Tha_L_8_1	0.024 ± 0.027	0.019 ± 0.027	0.021 ± 0.026	0.025 ± 0.031	0.019 ± 0.025
Tha_R_8_1	0.016 ± 0.018	0.020 ± 0.023	0.018 ± 0.025	0.019 ± 0.026	0.018 ± 0.025
Tha_L_8_2	0.009 ± 0.016	0.010 ± 0.021	0.010 ± 0.023	0.008 ± 0.018	0.011 ± 0.024
Tha_R_8_2	0.014 ± 0.021	0.011 ± 0.019	0.008 ± 0.017	0.008 ± 0.020	0.008 ± 0.017
Tha_L_8_3	0.014 ± 0.018	0.013 ± 0.021	0.012 ± 0.019	0.010 ± 0.019	0.010 ± 0.018
Tha_R_8_3	0.011 ± 0.022	0.008 ± 0.017	0.011 ± 0.017	0.009 ± 0.018	0.013 ± 0.017
Tha_L_8_4	0.033 ± 0.026	0.028 ± 0.025	0.029 ± 0.029	0.025 ± 0.027	0.031 ± 0.032
Tha_R_8_4	0.029 ± 0.029	0.025 ± 0.028	0.023 ± 0.029	0.025 ± 0.029	0.020 ± 0.029
Tha_L_8_5	0.027 ± 0.025	0.032 ± 0.029	0.027 ± 0.031	0.025 ± 0.027	0.025 ± 0.028
Tha_R_8_5	0.024 ± 0.024	0.020 ± 0.021	0.018 ± 0.023	0.021 ± 0.025	0.015 ± 0.020
Tha_L_8_6	0.018 ± 0.023	0.017 ± 0.024	0.021 ± 0.024	0.021 ± 0.023	0.020 ± 0.026
Tha_R_8_6	0.024 ± 0.023	0.020 ± 0.025	0.020 ± 0.023	0.017 ± 0.019	0.020 ± 0.025
Tha_L_8_7	0.023 ± 0.025	0.023 ± 0.027	0.021 ± 0.024	0.016 ± 0.023	0.020 ± 0.023
Tha_R_8_7	0.022 ± 0.026	0.017 ± 0.021	0.016 ± 0.022	0.017 ± 0.026	0.014 ± 0.020
Tha_L_8_8	0.024 ± 0.027	0.020 ± 0.024	0.021 ± 0.026	0.018 ± 0.022	0.022 ± 0.027
Tha_R_8_8	0.022 ± 0.023	0.021 ± 0.022	0.023 ± 0.026	0.024 ± 0.028	0.024 ± 0.026
Note. Mean ± SD, not adjusted for age, sex, and scanner.					
